# Shear-banding Induced Indentation Size Effect in Metallic Glasses

**DOI:** 10.1038/srep28523

**Published:** 2016-06-21

**Authors:** Y. M. Lu, B. A. Sun, L. Z. Zhao, W. H. Wang, M. X. Pan, C. T. Liu, Y. Yang

**Affiliations:** 1 Institute of Physics, Chinese Academy of Sciences, Beijing 100190, P.R. China; 2 Centre for Advanced Structural Materials, Department of Mechanical and Biomedical Engineering, City University of Hong Kong, Tat Chee Avenue, Kowloon Tong, Kowloon, Hong Kong SAR, P.R. China

## Abstract

Shear-banding is commonly regarded as the “plasticity carrier” of metallic glasses (MGs), which usually causes severe strain localization and catastrophic failure if unhindered. However, through the use of the high-throughput dynamic nanoindentation technique, here we reveal that nano-scale shear-banding in different MGs evolves from a “distributed” fashion to a “localized” mode when the resultant plastic flow extends over a critical length scale. Consequently, a pronounced indentation size effect arises from the distributed shear-banding but vanishes when shear-banding becomes localized. Based on the critical length scales obtained for a variety of MGs, we unveil an intrinsic interplay between elasticity and fragility that governs the nanoscale plasticity transition in MGs. Our current findings provide a quantitative insight into the indentation size effect and transition mechanisms of nano-scale plasticity in MGs.

Shear banding is the major mechanism of deformation to accommodate plasticity in metallic glasses (MGs)^1^,^2^,^3^,^4^. As a result of shear softening, shear-banding usually manifests as severe strain localization, causing catastrophic failure if unhindered^5^,^6^. This is in sharp contrast to dislocation-mediated plasticity in crystalline metals, in which dislocation slip lines could multiply, interact and form complex entanglements giving rise to homogeneous-like plastic deformation across a wide range of length scales^7^. The dislocation nature of plasticity enables crystalline metals to strain harden and renders them with a prominent strength size effect^7^, that is, the smaller the stronger. From the application perspective, understanding the small-scale plasticity in crystalline metals is of great importance to the design and fabrication of crystalline-metal based micro- and nano-structures, which have hence been well studied over the past decades^7^,^8^.

On the other hand, MGs are also an excellent candidate structural material for fabricating micro/nano-devices^9^,^10^. By exploiting the super-plasticity of MGs in their super-cooled liquid regime, it was demonstrated that a variety of MG-based micro- and nano-structures with complex shapes could be readily made^9^,^11^. This opens up a new route to the fabrication of small-scale structures and brings about opportunities to advance the current technology of micro-electro-mechanical systems (MEMS) that was used to be based on crystalline materials. Nevertheless, fundamental issues also arise, one of which is related to whether small-sized MGs would behave similarly as the large-sized ones under mechanical loadings. Since shear banding is the “plasticity carrier” of MGs^1^,^2^,^3^,^4^, this is equivalent to ask: how do shear bands behave at the small size scale in comparison to those at the large scale? Over the past years, there were tremendous efforts that have been dedicated to addressing this important question^12^,^13^,^14^. However, till today, the issue of size effect on shear banding in MGs is yet to be fully settled, which still remains actively discussed and intensely debated in the literature^7^,^15^,^16^,^17^.

In the classic studies of materials size effect, indentation has been remaining as one of the most efficient tools to probe the mechanical behavior of materials of an extremely small size^13^,^18^,^19^. Compared to the micro- and nano-compression techniques, indentation does not entail a complicated process for preparing small-sized samples, such as the use of focused ion beam (FIB) to fabricate micro-/nano-pillars for testing, during which process surface defects might be introduced, thus altering the mechanical behaviors of the micro-samples so obtained^20^,^21^,^22^. This is particularly so for MGs as noted in a few recent studies^21^,^22^. According to Magagnosc *et al*.^21^, FIB-milled MG micropillars appear stronger but less ductile than the micropillars obtained through hot embossing molding. Furthermore, through controlled surface ion-implantation, it was also demonstrated that nano-scale plasticity of MGs can be tuned by adjusting the dose of the implanted ions^22^,^23^. In view of these recent findings^20^,^21^,^22^,^23^, one can conclude that the behavior of nano-scale shear-banding in MGs relies on how micro-samples are prepared. Hence, the issue of size effect on shear-banding in pristine MGs is not completely settled and more research efforts are still needed. Unlike the previous studies^18^,^23^,^24^,^25^, here we choose indentation as the experimental means to study the size effect on shear banding in MGs. By doing so, we can avoid any “complexities” that may result from the process of micro-sample preparation. Meanwhile, we can also study the possible size effect on shear banding in the presence of a stress concentrator, which is related to the physical origin of high fracture toughness in MGs^26^ but received much less attention in the past years.

## Results

### Dynamic Nanoindentation Analysis

For the current study, six MGs were prepared via melt-spinning (see Method), including Cu_46_Zr_46_Al_8_, Zr_52.5_Cu_17.9_Ni_14.6_Al_10_Ti_5_, Pd_40_Cu_30_Ni_10_P_20_, Mg_65_Cu_25_Gd_10_, Ce_60_Al_20_Ni_10_Cu_10_ and La_60_Ni_15_Al_25_ (in atomic percentage). The structural amorphousness of the MGs was confirmed via X-ray diffraction (XRD) (see Supplementary Fig. 1) and DSC (see Supplementary Fig. 2). Subsequently, dynamic nanoindentation tests with a Berkovich indenter were performed on the pristine surfaces of the as-spun MG ribbon samples, which exhibit low roughness. For each sample, at least six indents were reduplicated to verify the validity and scatter of the indentation data. Unlike regular nanoindentation tests in which only a quasi-static load is applied, a secondary sinusoidal cyclic load is superimposed onto a primary quasi-static load in our dynamic nanoindentation test, which enables us to extract continuously the instantaneous hardness as well as elastic modulus of the testing material as a function of the indentation displacement (see Method). This capacity of high-throughput data acquisition facilitates the monitoring of the hardness variation in the MGs in the early stage of shear-banding. To avoid a possible strain rate effect on shear banding in previous studies^27^,^28^,^29^, we fixed the indentation strain rate, as keyed to the primary load function (see Supplementary Fig. 3), to be a constant of about 0.01 s^−1^ throughout the whole tests.

**Figure 1:**
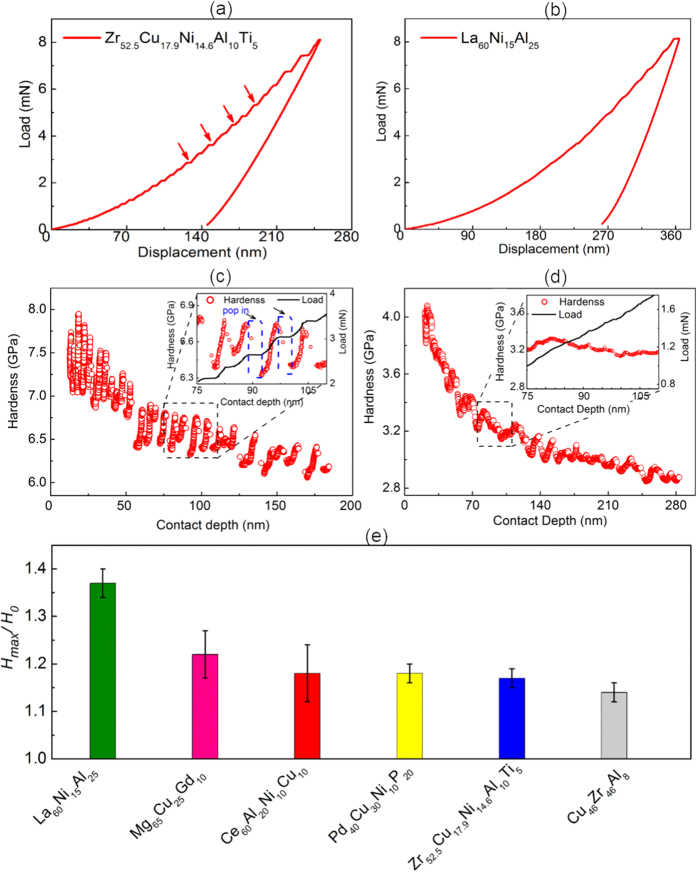
Experimental results obtained in the dynamic indentation tests. (**a**,**b**) are typical load-displacement (*P-h*) curves for Zr- and La-based MG obtained under a constant strain rate at room temperature. Red circles in (**c**,**d**) are corresponding nanohardness data for Zr- and La-based MG obtained through *P-h* curve. The inset shows the enlarged part of the hardness data (red) and the corresponding *P-h* curves (black). (**e**) shows the average values of *H*_*max*_*/H*_*0*_ for different MGs in six measurements. *H*_*0*_ and *H*_*max*_ represent bulk hardness and hardness value at the indentation depth of 30 nm, respectively.

Figure 1(a,b) show respectively the typical load-displacement (*P-h*) curves for the Zr- and La-based MG ribbons obtained in our dynamic indentation tests with the peak load of 0.8 mN. Evidently, the *P-h* curve of the Zr-based MG is serrated and exhibits multiple prominent displacement “pop-ins”, as indicated by the arrows in Fig. 1(a); in contrast, the *P-h* curve of the La-based MG appears relatively smooth. A similar phenomenon of serrated plastic flow was also observed in the indentation of other MGs (see Supplementary Fig. 4); however, the extent of load serration (or displacement pop-in) varies with the chemical composition of the MG. From the dynamic indentation, the instantaneous hardness *H* can be obtained point-by-point along the *P-h* curve. As shown in Fig. 1(c,d), both the Zr- and La-based MGs exhibit a general trend of indentation size effect, namely, the hardness *H* increases with the decreasing indentation depth *h*. However, unlike the regular indentation size effect in crystalline metals, which manifests as a monotonic increase in hardness^8^,^30^, the *H-h* curve of the MGs appears “serrated”. In most cases, the hardness strongly fluctuates around the general trend of indentation size effect [Fig. 1(c)]. As shown by the inset of Fig. 1(c), a close examination of the experimental data clearly reveals a one-to-one correspondence between the hardness drop and the displacement pop-in. After the cessation of pop-in, the hardness immediately increases. This apparent post-pop-in “elastic” deformation could involve hidden processes of anelasticity, as recently revealed by the nano-electric-contact-resistance measurement^31^. Similar “serrated” *H-h* curves were also observed in other MGs (see Supplementary Figs 5 and 6). However, it is worth noting that the *H-h* curve of the La-based MG appears relatively “smooth”, as shown in Fig. 1(d) and the inset. Additionally, to ensure that these interesting observations were not affected by the superimposed secondary dynamic loading, we also performed normal nanoindentation tests to obtain the MG hardness at different indentation depths. Our results show that the general trends of hardness versus indentation depth obtained from both methods are in excellent agreement (see Supplementary Fig. 7).

### Indentation Size Effect

As shown in Fig. 1(c,d) and Supplementary Fig. 5, our tested MGs all exhibit an indentation size effect. On one hand, as the indentation depth increases, the hardness decreases and approaches an asymptotic value *H*_*0*_ of bulk hardness, which should roughly equals three times the yield strength of the respective MGs. On the other hand, the rate of the hardness increase with the decreasing indentation depth varies. For the sake of comparison, here we define *H*_*max*_ to be the hardness at the indentation depth of 30 nm and *H*_*0*_the average hardness at the maximum indentation load of 8 *mN* at six measurements, and then use the ratio of *H*_*max*_*/H*_*0*_ to differentiate the indentation size effect in different MGs (see Supplementary Table 1). As seen in Fig. 1(e), Supplementary Fig. 8, the La-based MG displays the “strongest” size effect with *H*_*max*_*/H*_*0*_ ≈ 1.37 while the Cu-based MG the “weakest” with *H*_*max*_*/H*_*0*_ ≈ 1.14. These variations of the value (*H*_*max*_*/H*_*0*_) and fluctuations of hardness, as coming along with the indentation size effect in MGs, are in sharp contrast to the well-behaved and smooth indentation-size-effect in crystalline metals^8^, indicating a fundamentally different mechanism of nano-scale plasticity as compared to that in crystalline metals. Consequently, this defies the direct use of the conventional indentation-size-effect model, such as that based on strain gradient plasticity^32^, for the interpretation of our experimental data.

**Figure 2:**
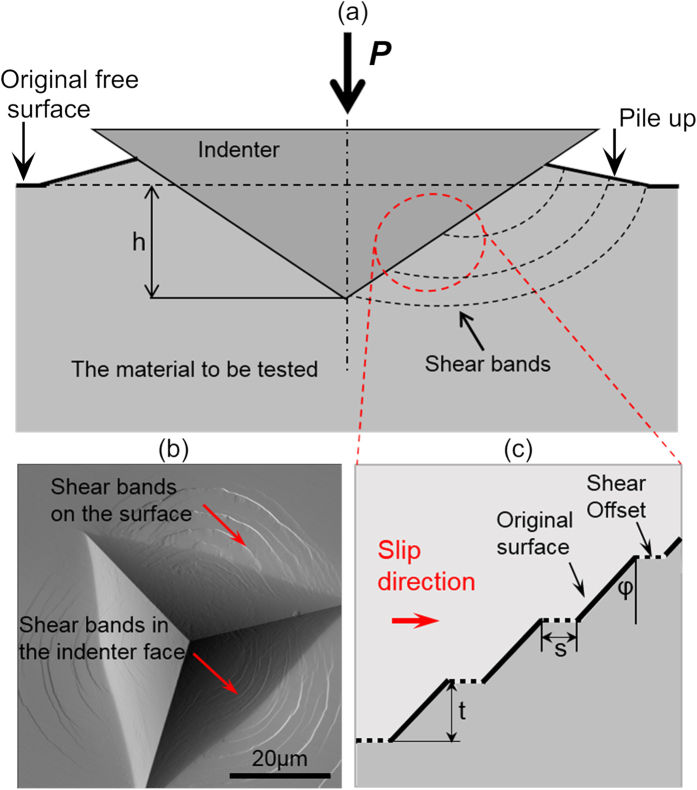
Schematic illustration of the DS model. (**a**) The plastic flow beneath a Berkovich indenter. *P* is indentation load and *h* is indentation depth. (**b**) Configuration of shear band structures around the indent, illustrating a series of semicircular shear band traces on the surface and in the indenter face. The illumination-mode AFM image is obtained in Pd_40_Cu_30_Ni_10_P_20_ ribbons at the indentation load of 8 N. (**c**) An enlarged part of the contact surface in (**a**). Shear bands in the indenter face are idealized as triangular shear band loops with slip direction and shear offsets parallel to the original free surface.

To quantitatively understand the underlying physical mechanisms, here we develop a “discrete shear-band” (DS) model to capture the general trend of the indentation size effect in MGs. As illustrated in Fig. 2(a), our DS model is based on the fact that an indentation impression in MGs comprises numerous discrete shear bands emanating from the tip-surface interface [Fig. 2(b)]. For the Berkovich indentation, the shear-band geometries can be simplified as triangular loops situated along the indentation depth *h* with an average spacing *t*, as illustrated in Fig. 2c. As a result, mechanical energies are dissipated in the initiation/propagation of different-sized shear bands, the stretching of existing surfaces between shear bands, creation of new surfaces and bulk plastic deformation. In theory, we can write the general equation of energy balance as follows:


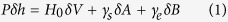


where *P* is the indentation load, *δh* the incremental indentation displacement, *δV* the incremental bulk volume plastically deformed underneath the indenter, *δA* the incremental change in the area being stretched between adjacent shear bands, *γ*_*s*_ the surface stress required to stretch/displace this incremental area from flat to inclined plane, *δB* the incremental shear-offset area newly generated by the sliding of a shear band and *γ*_*e*_ the total energy spent per unit area involved in initiating an autocatalytic growth of a shear band as discussed in refs 33 and 34. Thus, *H*_0_*δV* stands for the energy for propagating shear bands throughout the sample volume, namely bulk plastic deformation, *γ*_*e*_*δB* for the energy for initiating shear bands, and *γ*_*s*_*δA* for the energy for stretching the existing surfaces between adjacent shear bands. Here, it is worth noting that friction effect didn’t enter into the equation for hardness because of the negligible effect on hardness measurement for the blunt Berkovich tip used in the current study^35^,^36^. Meanwhile, given the generality of the energy balance principle, similar equations as equation (1) were also derived in the nanoindentation study of other types of materials^30^,^37^. According to the reported works^33^,^38^, it was estimated that γ_*s*_ ~ 1 J/m^2^ and γ_*e*_ ranges from 100 to 10000 J/m^2^ for MGs. As such, the following equation can be derived based on equation (1) for the hardness of MGs (see Supplementary Note 1):


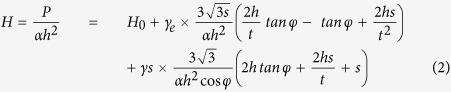


where *s* denotes the average shear offset, *α*(=24.5) is a constant geometric factor for a Berkovich indenter and *φ* is the angle as shown in Fig. 2(c). Here it should be noted that, based on the recent work of Zhang *et al*.^39^, the average shear-band spacing *t* can be related to the material density, the shear wave velocity and the yield strength of MGs, which can be estimated for different MGs (see Supplementary Note 2).

**Figure 3:**
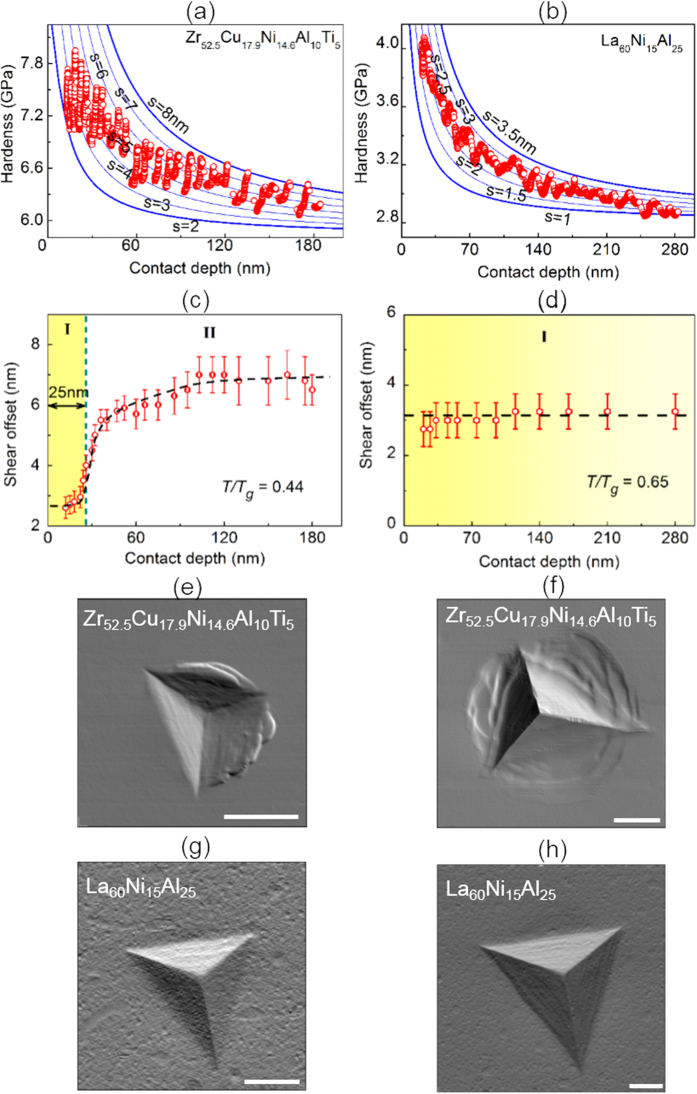
The fitting results of the DS model for Zr- and La-based MG. (**a**,**b**) show the experimental hardness data (red circles) and theoretical fitting (blue lines) of the DS model, in which *s* represents the value of shear offset. (**c**,**d**) display the depth dependence of shear offset of the fitting results shown in (**a**,**b**). *T* and *Tg* are room temperature and glass transition points, respectively. The black dotted lines are drawn for eye guides. (**e**–**h**) are illumination-mode AFM images of indentation impression for Zr- and La-based MGs at indentation load of 2 mN and 8 mN. The scale bars are 500 nm.

By fixing γ_*s*_ around ~1 J/m^2^ and *H*_*0*_ to be the bulk hardness for the respective MGs, we can fit our experimental data of *H* versus *h* with our DS model by treating γ_*e*_ and *s* as the fitting parameters. As shown in Fig. 3(a,b), it can be seen that the general trend can be well captured by our model by taking γ_*e*_ ~ 100 J/m^2^; however, because of the hardness fluctuation, the exact matching of the experimental data with the DS model is not possible unless the appropriate value for the shear offset *s* is selected. In data processing, we first obtained γ_*e*_ by fitting the DS model to the general trend of our experimental data and obtained *s* afterwards by matching the model prediction with the measured hardness. By doing so, we can obtain the shear offsets at the different indentation depths, as shown in Fig. 3(c,d). Based on our fitting results, the following interesting trends unfold: (i) the shear offset *s* tends to increase with the indentation depth *h*, particularly so for a deep indentation (*the indentation size effect on shear offset*) [Fig. 3(c)]; and (ii) a strong indentation size effect on hardness *H* usually corresponds to a weak size effect on the shear offset and *vice versa*. Here it should be emphasized that, to test the robustness of our model fitting, the value of γ_*s*_ was also varied by two orders of magnitude. However, the results show that good fitting can be only obtained when γ_*s*_ falls in the range between 1 and 10 J/m^2^ (see Supplementary Fig. 10). Within this range of γ_*s*_, the fitted values of γ_*e*_ and *s* are not sensitive to the value of γ_*s*_.

To further verify the validity of our modeling, atomic force microscopy (AFM) was utilized to measure the shear offsets exposed on the sample surfaces at different indentation depths (see Supplementary Fig. 11). According to the AFM results, the measured shear offsets show a similar trend of size effect as our theoretical model unveils, that is, the deeper and wider is the indent the larger is the average shear offset, as exemplified in Fig. 3(e,f) and the cross-sectional AFM profiles in Supplementary Fig. 12. However, we should emphasize that the different-sized indents on the La-based MG all appear quite “smooth” without a clear trace of individual shear offsets, as seen in Fig. 3(g,h). Even though clear slip steps cannot be seen in AFM images, the curved edges of indentation impression, as shown in Fig. 3h, is a common feature of shear band formation and at the same time, pile-up around the indenter also provides the strong evidence for shear bands^25^ (see Supplementary Fig. 13). Meanwhile, shear bands instead of microcracks can be observed at the indentation load as large as 80 mN (see Supplementary Fig. 14). This implies that the shear offsets in the La-based MG at the small indentation load are very small, which can’t be detected in our AFM setting, and do not vary too much with the indentation depth, which agrees very well with our theoretical prediction [Fig. 3(d)]. Given such a small shear offset, it can be envisioned that a large density of shear bands is demanded to accommodate the significant plastic flow caused by indentation. This is analogous to a scenario of “distributed” shear-banding, for which multiple shear bands with small shear offsets operate simultaneously, as exemplified by the relatively smooth indentation profiles on the surface of the La-based MG [Fig. 3(g,h)]. In contrast, shear bands are “localized” if shearing is mainly concentrated on one or a few shear bands. This results in the clear traces of shear offsets that increase with the indentation depth, as shown by the indent profiles on the surface of the Zr-based MG [Fig. 3(e,f)].

### Size-Controlled Plasticity Transition

**Figure 4:**
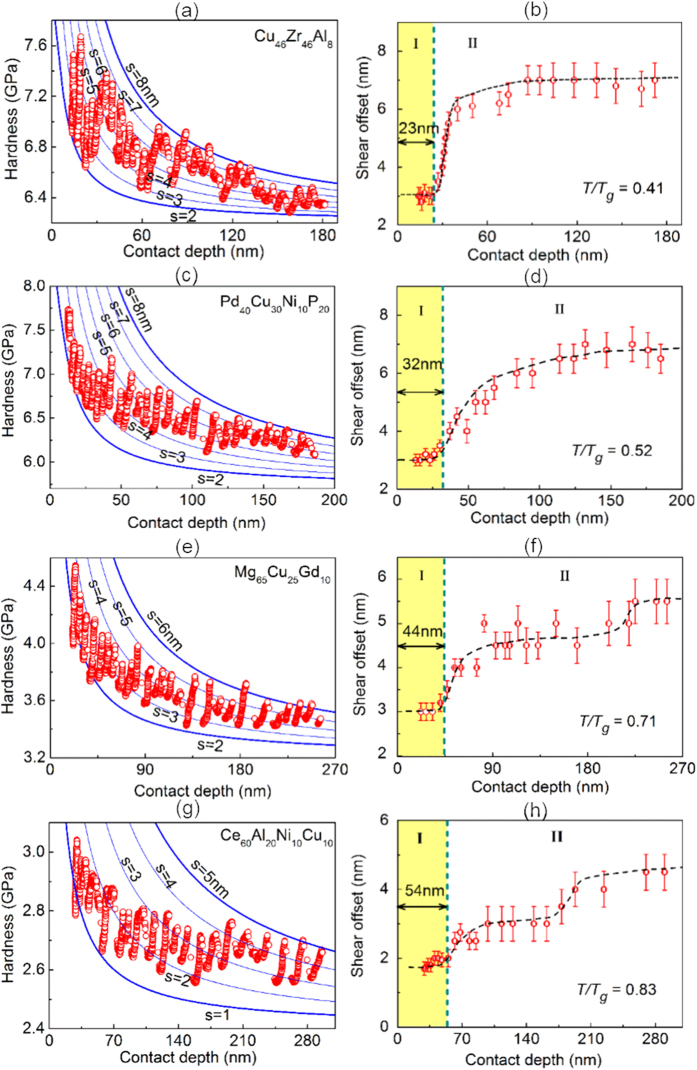
The fitting results of the DS model for different MGs. The left figures show the experimental hardness data (red circles) and corresponding theoretical fitting (blue lines) of the DS model. The right figures are depth dependence of shear offset of the fitting results in the left figures. The black dotted lines are drawn for eye guides.

Based on the obtained shear offsets, we can now differentiate the different types of indentation plastic flows in MGs and classify them into two regimes: Regime I corresponds to a constant shear offset or “distributed shear banding” while Regime II to a varying shear offset or “localized shear banding”. As shown in Fig. 4(a–h), one can clearly see a general trend for a size-controlled transition from distributed to localized shear banding, within the range of the indentation depth from ~15 nm to ~300 nm, for different types of MGs, which is similar to the behavior of Zr-based MG [Fig. 3(c)]. At the very small indentation depth, the shear offsets remain more or less a constant, indicative of distributed shear banding (Regime I). In this regime, the hardness rises sharply with the decreasing indentation depth. By comparison, when the indentation depth exceeds a critical length scale *l*_*cr*_, there is a significant increase in the shear offset and the plastic flow transits into Regime II. In this regime, it is the hardness rather than shear offset that remains to be a constant. The critical length scale *l*_*cr*_ for the plasticity transition can be obtained for different MGs, which however varies with the chemical composition of the material, as summarized in Table 1. Here, it is worth noting that it seems that more than two transitions could be observed in Fig. 4(f–h). At the current moment, we still do not understand the reason for such a weird behavior. This demonstrates that the real dynamics of shear banding could be very complicated because of its statistic nature. However, since most MGs we studied only exhibit one transition, the transition we defined refers to the first one that is common to all MGs. According to our results, the smallest critical length is found to be *l*_*cr*_ ~ 20 nm for the Zr-based MG [Fig. 3(c)] and the Cu-based MG [Fig. 4(b)]. By comparison, the critical length is estimated to be greater than ~280 nm for the La-based MG [Fig. 3(d)]. Similar strain rate-controlled transition behavior of shear banding has also been reported in refs 27 and 29, however, we should note that our work is different from previous works because we studied the size-controlled effect on the transition behavior of shear banding while the characteristic strain rate was kept at a constant.

## Discussion

With the dynamic nanoindentation results and the suggested theoretical modeling, we have uncovered size-controlled plasticity transition through the indentation of different MGs. Now we would like to discuss the important implications of our results and the physical origins of the observed size effects. First, let us discuss the average dissipation energy *γ*_*e*_ ~ 100 J/m^2^ as obtained from our data fitting for shear-band formation in MGs (see Supplementary Table 1). Compared to the typical surface energies (0.5–10 J/m^2^) of metals^40^, the value of the obtained *γ*_*e*_ is very high, which implies that shear-band formation involves not only surface creation but also other dissipative processes. Based on thermodynamics, it has been shown that the stress-induced shear-band formation is akin to a process of stress driven glass-to-liquid transition^41^,^42^. In such a case, *γ*_*e*_ includes the activation energy for glass transition, which should be much larger than the nominal surface energy. Furthermore, within the framework of the shear-transformation-zone (STZ) model, it was shown by Jiang *et al*.^33^ that this shear-band formation energy should be on the order of 10^2^–10^4^ J/m^2^, which is consistent with our current results.

**Figure 5:**
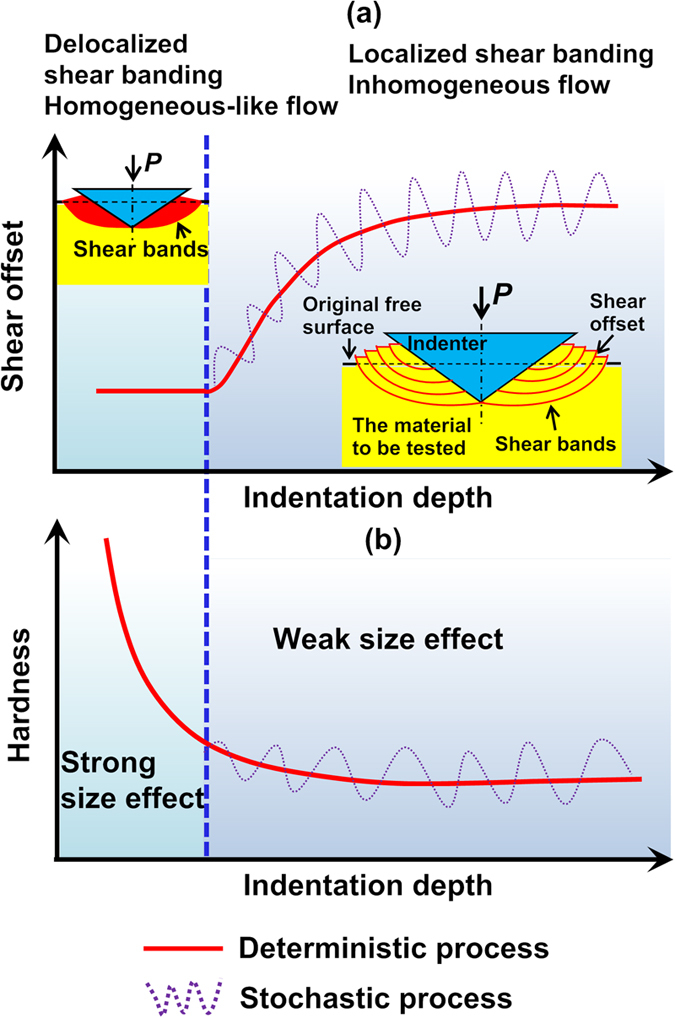
Size effect on hardness and shear offset. (**a**,**b**) illustrate the evolution of shear offset and hardness with increasing indentation depth.

Next, let us discuss the physical origin of the observed size effects in the MGs. As previously discussed, there are two complementary size effects (hardness and shear offset) showing the opposite trend. As illustrated in Fig. 5, shear banding is distributed for shallow indentation, which corresponds to a constant shear offset or a scenario of multiple shear banding, while the hardness increases with the decreasing indentation depth. This behavior is analogous to the indentation size effect observed in crystalline metals for which each dislocation slip band produces the same amount of shear offset^43^. As a result of the large area-volume ratio at small indents, it is known that hardness can increase significantly in the crystalline metals because of the enhanced dissipation of mechanical energies^37^. In line with the DS model we developed, this also serves as the mechanism for the observed size effect on the hardness of the MGs. To validate this thinking, we also applied our DS model to fit the indentation results of a single crystal Ni, which show significant indentation size effect as our La-based MG (see Supplementary Fig. 15). The fitting results indicate a constant shear offset, agreeing with the fact that each “shear band” here which represents a dislocation slip line produces the same amount of shear offset and there is no transition in the plastic flow mechanism in the single crystal Ni. On the other hand, shear banding is localized for deep indentation and longer shear bands tend to produce greater shear offsets in a statistic sense. As this occurs, the associated hardness remains a constant [Fig. 5]. When the indentation deepens, the area-volume ratio reduces and thus the hardness approaches its bulk value. As for the observed size effect on the shear offset, a similar phenomenon was also observed in uniaxial testing of MGs^44^,^45^. In view of the similarity, we can rationalize the size effect on the shear offset from two aspects: (1) due to shear localization , there is a dimensional misfit between the planar energy dissipation and volumetric energy release, which can therefore result in the size effect on the shear offset^44^; (2) if the propagation of the localized shear bands in indentation can be viewed as a stick-slip process, longer shear bands, which commonly correspond to higher sample stiffness, also causes larger shear offsets because of the intrinsic size effect in stick-slip as already discussed in ref. 45. Note that a similar finding was also discovered and explained by Chen *et al*.^15^,^46^ even without using the stick-slip model.

**Figure 6:**
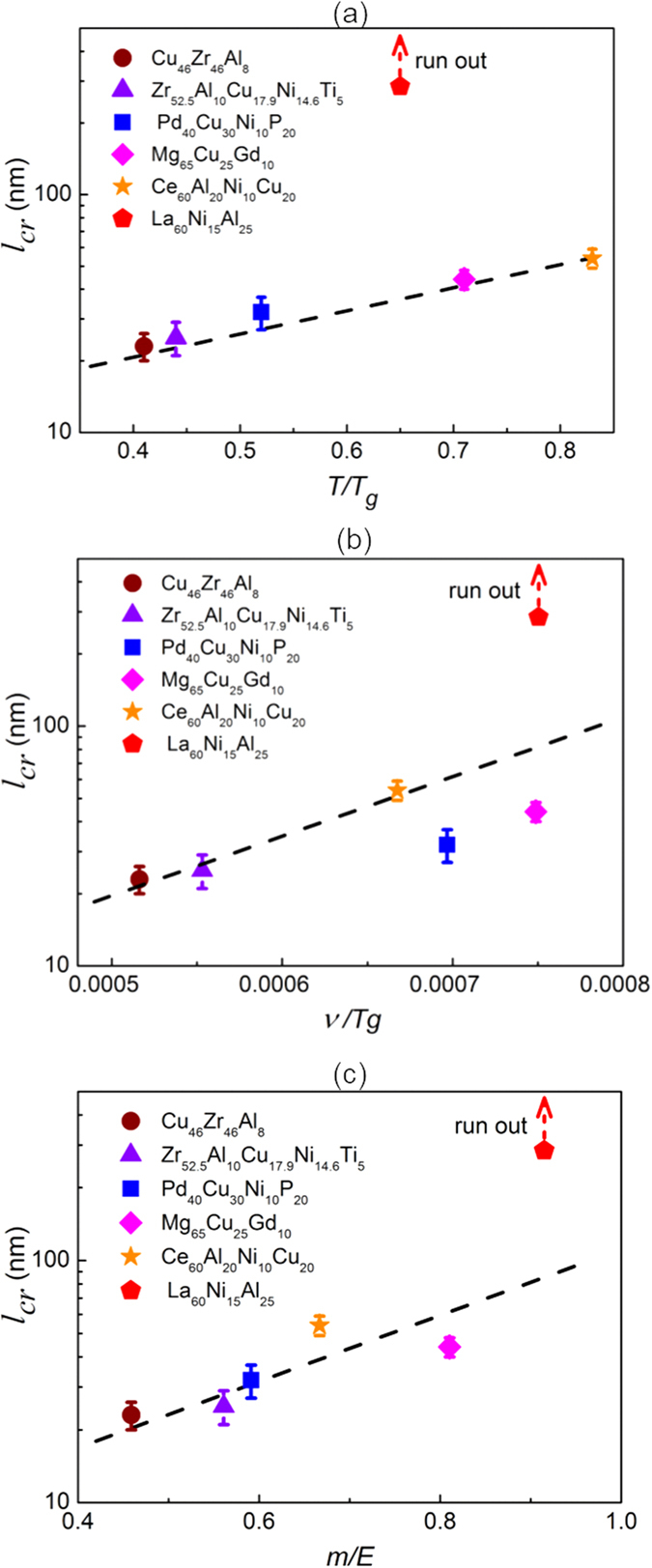
Correlation between the elasticity (and fragility) and the critical depth of the six MGs. *T*_*g*_, ν, *m* and *E* represent glass transition point, Poisson’s ratio, fragility and elastic modulus of MGs. The black dotted lines are drawn for eye guides.

Now one may ask what controls the critical length that characterizes the transition from distributed to localized shear banding in the MGs. Since it is known that plastic flow in MGs is homogenous above the glass transition point *T*_*g*_ while transits into the inhomogeneous flow or shear banding only at the low temperatures^1^,^2^,^3^,^4^, one natural response to the above question is that the plasticity transition should be related with the glass transition point *T*_*g*_ of the individual MGs^47^. Figure 6(a) shows the plot of the critical length scale *l*_*cr*_ versus the homologous temperature (*T/T*_*g*_) for the various MGs we studied. Evidently, there is a clear correlation between *l*_*cr*_ and *T/T*_*g*_ for most MGs except for the La-based MG. The correlation established for the many MGs indicates that the glass transition point (*T*_*g*_) is likely to play an important role; however, the departure of the data from the La-based MG from the trend also suggests that the glass transition point may not be the sole factor that determines the behavior of plasticity transition.

According to refs 34 and 48, shear banding in MGs is essentially a process of mechanical instability being triggered by stress-induced softening. It has been also shown by atomistic simulations^49^ and experiments^34^ that the localized softening zone would undergo a subcritical stage at the small size scale with a rather slow growth rate^34^ and continue to grow under stress towards an autocatalytic critical stage that is characterized by a very rapid growth rate close to the sound speed^49^. From a mechanistic point of view, this transition from slow to rapid growth of the small-sized or embryonic shear band echoes the plasticity transition we here observed and is mainly controlled by two factors: one is the elastic constant *E* of the surrounding materials that serve as the elastic confinement of the growing shear-band embryo and the other is the softening rate in the shear-band embryo which is characterized by the stress loss per unit displacement^34^. Following this line of reasoning, it can be derived that the propensity for an autocatalytic shear-band growth in a MG is proportional to the ratio of the softening rate to the elastic constant^34^. Since shear banding is stress-induced glass transition from a thermodynamic viewpoint^41^,^42^, it can be hence inferred that the softening rate within a shear-band embryo should scale with the fragility, *m*, of the MG^50^. As such, we may envision that the ratio of *m/E* can be viewed as an index to quantify how easily a mature shear band can form in a MG: the higher is *m*/*E* the shorter is the critical shear band embryo size and thus, the easier is to produce a mature shear band. Based on the above thinking, we can therefore propose that the obtained critical length scale *l*_*cr*_ should scale with *m*/*E*. Since *m* scales with the Poisson’s ratio *v*^51^ and *E* with *T*_*g*_^52^, we should also have *l*_*cr*_ ~ *v/Tg*. To validate this proposal, we make a plot of *l*_*cr*_ versus *m*/*E* and *v/T*_*g*_ for the various MGs (see Supplementary Table 3). As shown in Fig. 6(b–c), one can see a clear positive correlation between *l*_*cr*_ and *m*/*E* or *v/T*_*g*_, which corroborates our idea that the plasticity transition observed across a wide range of chemical compositions is essentially a process controlled by the transition from slow to fast shear softening. In other words, the correlation length, which exhibits strong compositional dependence, can be regarded as an intrinsic reflection of nanoscale plasticity of MGs, similar to those macroscopic studies of serration dynamics^53^,^54^,^55^. Meanwhile, the annealing-induced structural evolution in MGs can also be detected through the change of *l*_*cr*_, the more stable the structure, the shorter the critical length (see Supplementary Note 3, Supplementary Table 4 and Supplementary Fig. 16). Therefore, the critical length scale *l*_*cr*_ is a measure of the easiness to initiate rapid shear softening and thus shear localization in various MGs. Finally, it is worth noting that a larger fragility index *m* is suggestive of a glassy structure more susceptible to local glass-to-liquid transition^56^, which is hence more likely to exhibit pronounced β-relaxation in mechanical spectroscopy^57^, which correlates with nanoscale strucutural heterogeneity^58^; therefore, our results also imply that the large critical length scale in the La-based MG should correspond to a pronounced β-relaxation, which can be also confirmed experimentally (see Supplementary Fig. 17).

In summary, a transition behavior of nanoscale plasticity is unveiled in our study through the systematic dynamic indentation tests, which shows that evolution of shear bands undergoes the transition from the “distributed” to localized mode. In the distributed regime, multiple shear bands with a constant but small shear offset are activated to sustain plastic strain, while in the localized regime one or a few dominant shear bands with large and varying shear offsets prevail. As a result of the enhanced energy dissipation in the distributed shear-banding regime, an indentation size effect on hardness becomes evident within a critical indentation depth. The observed critical length scale (*l*_*cr*_) in different MGs exhibits a strong correlation with the ratio of fragility (or Poisson’s ratio) to the elastic modulus (or the glass transition point). This important finding is consistent with the long standing notion that a high Poisson’s ratio is in favor of plasticity in MGs^59^. However, our results also show that plasticity in MGs is not only affected by the Poisson’s ratio but also intrinsically related to the dynamic and thermodynamic properties of the corresponding supercooled liquids, such as the fragility and glass transition point. Knowing this combined effect of elasticity and fragility can help us to have a better understanding of the transition mechanisms of nano-scale plasticity in MGs.

## Methods

### Sample preparation

Metallic ingots with nominal compositions of Cu_46_Zr_46_Al_8_, Zr_52.5_Cu_17.9_Ni_14.6_Al_10_Ti_5,_ Pd_40_Cu_30_Ni_10_P_20_, Mg_65_Cu_25_Gd_10_, Ce_60_Al_20_Ni_10_Cu_10_ and La_60_Ni_15_Al_25_ were prepared by arc-melting component elements with a purity of 99.9% or higher in a Ti-gettered ultrahigh purity argon atmosphere. Each master alloy was remelted several times to ensure a compositional homogeneity. To obtain glass ribbons, the melt-spinning method was used and the thickness of the ribbons is about 40 μm.

### Structural and dynamic characterization

The amorphous nature of the ribbons was confirmed by x-ray diffraction using Cu-K radiation in the Rigaku SmartLab X-Ray Diffractometer. The differential scanning calorimeter (DSC) measurements were conducted by using a TA #DSC Q20 at a heating rate of 20 K min^−1^. Atomic force microscope (AFM) characterization was performed to check the surface topography after nanoindentation tests using a non-contact Scan Asyst-Air (Bruker Dimension icon with Scan Asyst, USA) mode. The relaxation behaviors of these glass ribbons were characterized by dynamical mechanical spectroscopy (DMS) on a TA Q800 at a constant heating rate of 3 K min^−1^ with the frequency of 4 Hz. Additionally, the energy dispersive X-ray (EDX) characterization was also performed on the as-spun and oxidized La_60_Ni_15_Al_25_ ribbons (exposed in air for one month), as shown in Supplementary Fig. 18, which proves the negligible effect of oxidization at the as-spun state.

### Dynamic indentation tests

Nanoscale Dynamic Mechanical Analysis (nanoDMA III) measurements were performed on a Triboscope^TM^ Nanoindentation System (Hysitron, Minneapolis, MN, USA) with a Berkovich indenter at room temperature. It provides a truly continuous measurement of mechanical properties as a function of depth into a material’s surface^60^. During a nanoDMA test, a quasistatic force and a much smaller dynamic load at a user prescribed frequency is applied to the sample with the nanoindentation probe. The contact depth (*h*_*c*_) for the nanoDMA III analysis is calculated by 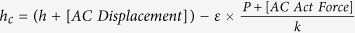, where *h* is the maximum displacement, [*AC Displacement*] is the dynamic displacement, *ε* is defined as a geometric constant (*ε* is 0.75 for the Berkovich indenter), *P* is the maximum force, [*AC Act Force*] is the dynamic force and *k* is the stiffness. The calculation of stiffness is based on the dynamic part through the equation of 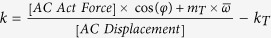, in which *φ* is the phase shift between the dynamic force and dynamic displacement, 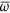 the radial frequency, *m*_*T*_ and *k*_*T*_ the mass and stiffness of the transducer, which can be obtained in the calibrations before tests. Hardness is solved with the maximum force (*P*) and contact area (*A*_*c*_) through 
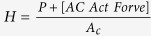
, in which *A*_*c*_ is the function of contact depth (*h*_*c*_). In this letter, the nanoDMA tests were performed approximately at a constant strain rate with the indentation load from 0 to 8 mN (see Supplemental Fig. 3), during which sinusoidal dynamic load with the frequency of 220 Hz was applied and the dynamic displacement in the range of 1–2 nm. Finally, it should be noted that, to ensure the data reproducibility, the areal function was calibrated to be valid within the range between 2 nm and 150 nm (see Supplementary Fig. 19).

## Additional Information

**How to cite this article**: Lu, Y. M. *et al*. Shear-banding Induced Indentation Size Effect in Metallic Glasses. *Sci. Rep.* **6**, 28523; doi: 10.1038/srep28523 (2016).

## Supplementary Material

Supplementary Information

## Figures and Tables

**Table 1:** Overview of the size effect on hardness and shear offset in various metallic glasses.

Composition	Size effect on hardness (*H*_*max*_*/H*_*0*_)	Size effect on shear offset	Critical length (nm)
Cu_46_Zr_46_Al_8_	Weak (1.14 ± 0.02)	Strong	23 ± 3
Zr_52.5_Cu_17.9_Ni_14.6_Al_10_Ti_5_	Modest (1.17 ± 0.02)	Modest	25 ± 4
Pd_40_Cu_30_Ni_10_P_20_	Modest (1.18 ± 0.02)	Modest	32 ± 5
Ce_60_Al_20_Ni_10_Cu_10_	Modest (1.18 ± 0.06)	Modest	54 ± 5
Mg_65_Cu_25_Gd_10_	Modest (1.22 ± 0.05)	Modest	44 ± 4
La_60_Ni_15_Al_25_	Strong (1.37 ± 0.03)	Weak	>280
