# Supplementary material for: Shear-banding Induced Indentation Size Effect in Metallic Glasses
Source: Sci Rep. 2016 Jun 21;6:28523. doi: 10.1038/srep28523 (PMC4914989; doi:10.1038/srep28523)
Supplement: Supplementary Information [file srep28523-s1.pdf]

Supplementary Information for "Shear-banding Induced Indentation Size Effect in Metallic Glasses"

Y. M. Lu[1,2], B.A. Sun[2], L. Z. Zhao[1], W. H. Wang[1], M. X. Pan[1,*], C.T. Liu[2], Y. Yang[2,*]

[1]Institute of Physics, Chinese Academy of Sciences, Beijing 100190, P.R. China.

[2] Centre for Advanced Structural Materials, Department of Mechanical and Biomedical Engineering, City University of Hong Kong, Tat Chee Avenue, Kowloon Tong, Kowloon, Hong Kong SAR, P.R. China

Correspondence and requests for materials should be addressed to Y. Y. (yonyang@cityu.edu.hk) and M.X. P. (panmx@aphy.iphy.ac.cn).

Supplemental materials including

Figs. S1-S19

Table S1-S4

Text S1-S3

References

Supplementary Figures

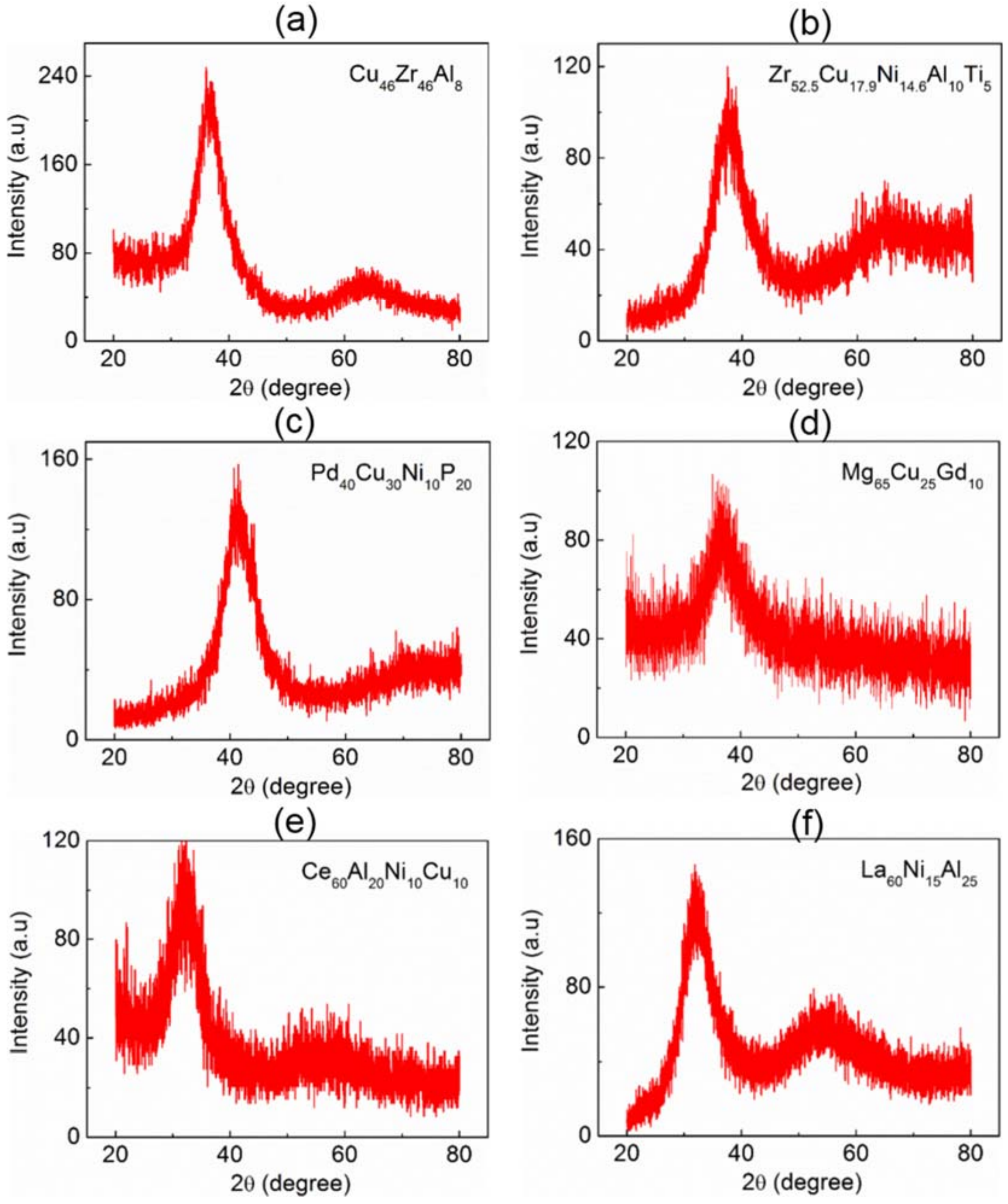

Supplementary Figure 1. X-ray diffraction patterns of the six MG ribbons.

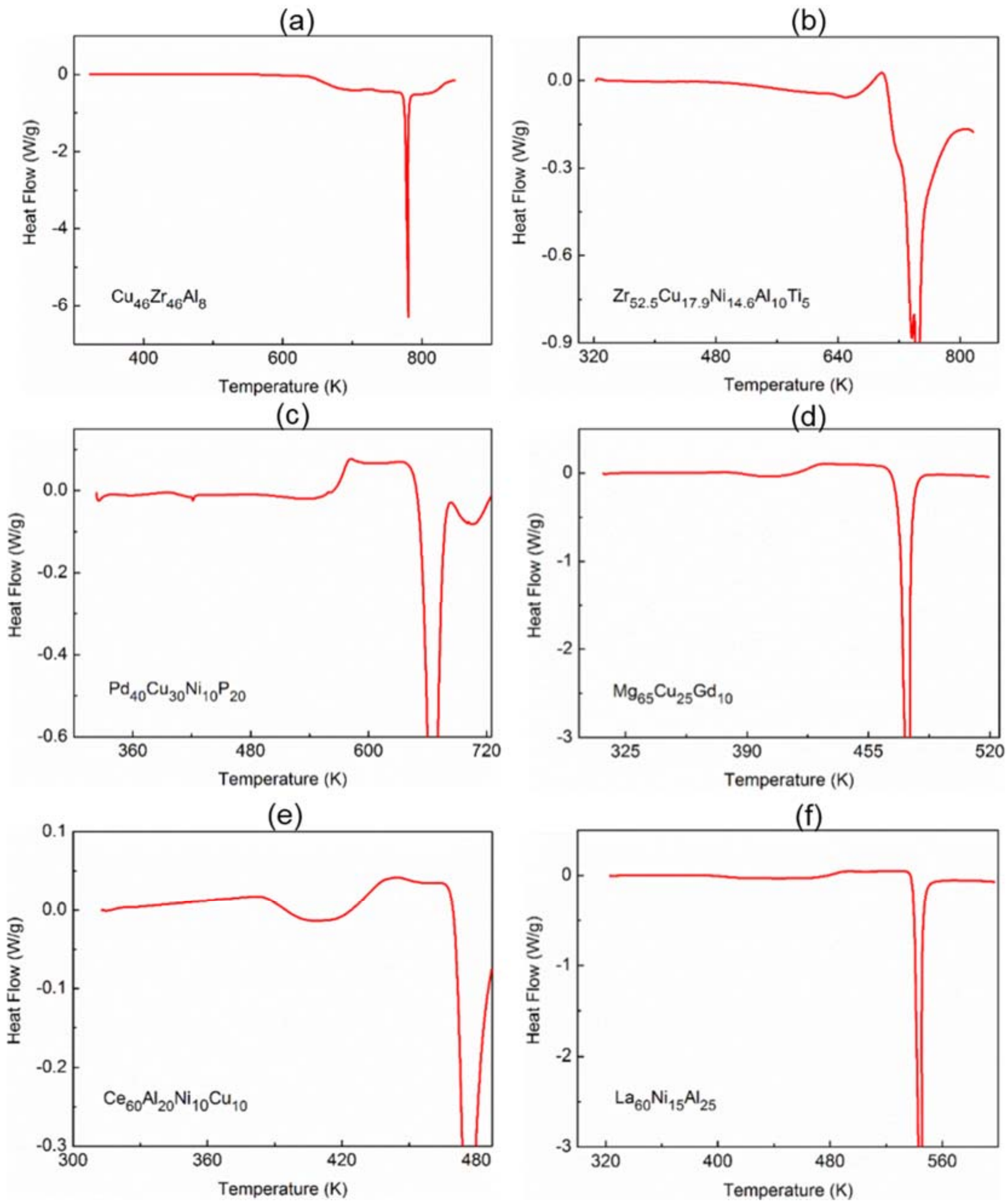

Supplementary Figure 2. The DSC curves of (a) $Cu_{46}Zr_{46}Al_8$, (b) $Zr_{52.5}Cu_{17.9}Ni_{14.6}Al_{10}Ti_5$, (c) $Pd_{40}Cu_{30}Ni_{10}P_{20}$, (d) $Mg_{65}Cu_{25}Gd_{10}$, (e) $Ce_{60}Al_{20}Ni_{10}Cu_{10}$ and (f) $La_{60}Ni_{15}Al_{25}$ MGs obtained at the heating rate of 20K min^{-1}.

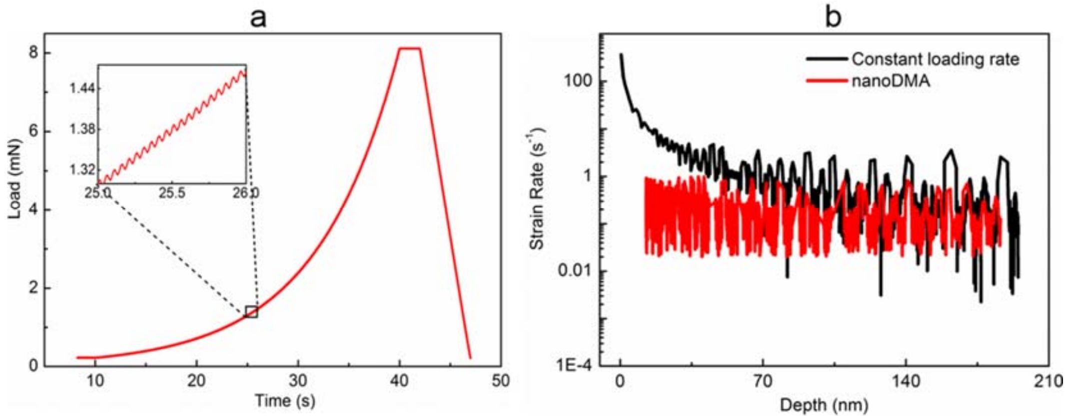

Supplementary Figure 3. (a) The time dependence of indentation load in nanoDMA tests for $Pd_{40}Cu_{30}Ni_{10}P_{20}$ MG. The insert is the enlarged part of the load-time curve and the dynamic sinusoidal characteristics can be clearly seen. (b) Strain rate versus indentation depth in nanoDMA and normal constant loading rate ($1.6 mN \cdot s^{-1}$) tests for $Pd_{40}Cu_{30}Ni_{10}P_{20}$ MG. Strain rate is calculated by using the equation: $\dot{\varepsilon} = \dot{h} / h$, in which h is the indentation depth and $\dot{h}$ is the rate of change in the indentation depth.

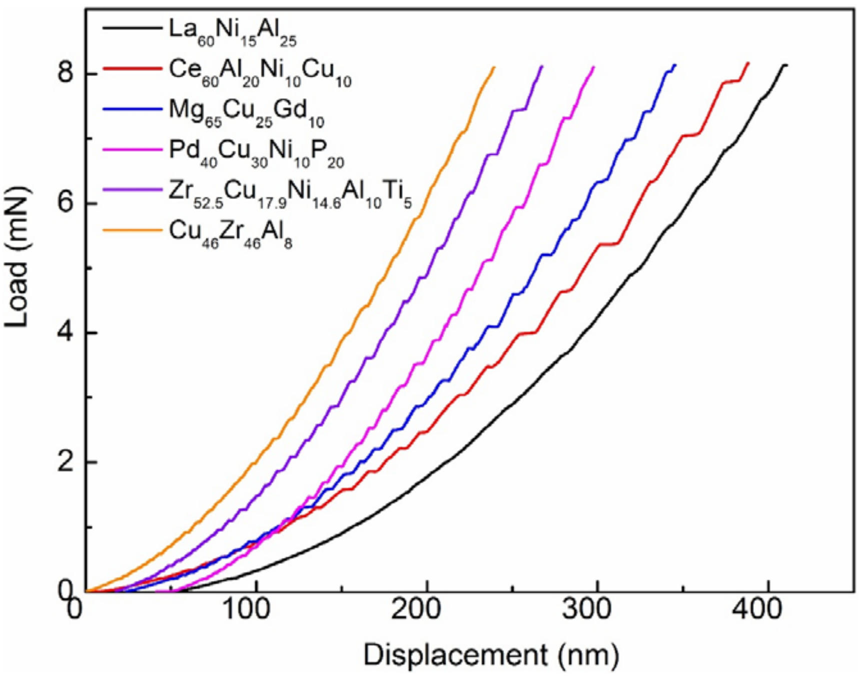

Supplementary Figure 4. Load-displacement curves for the six MGs obtained on the loading portion under constant strain rate at room temperature with the peak load of 8mN. Curves are offset from the origin for clear viewing.

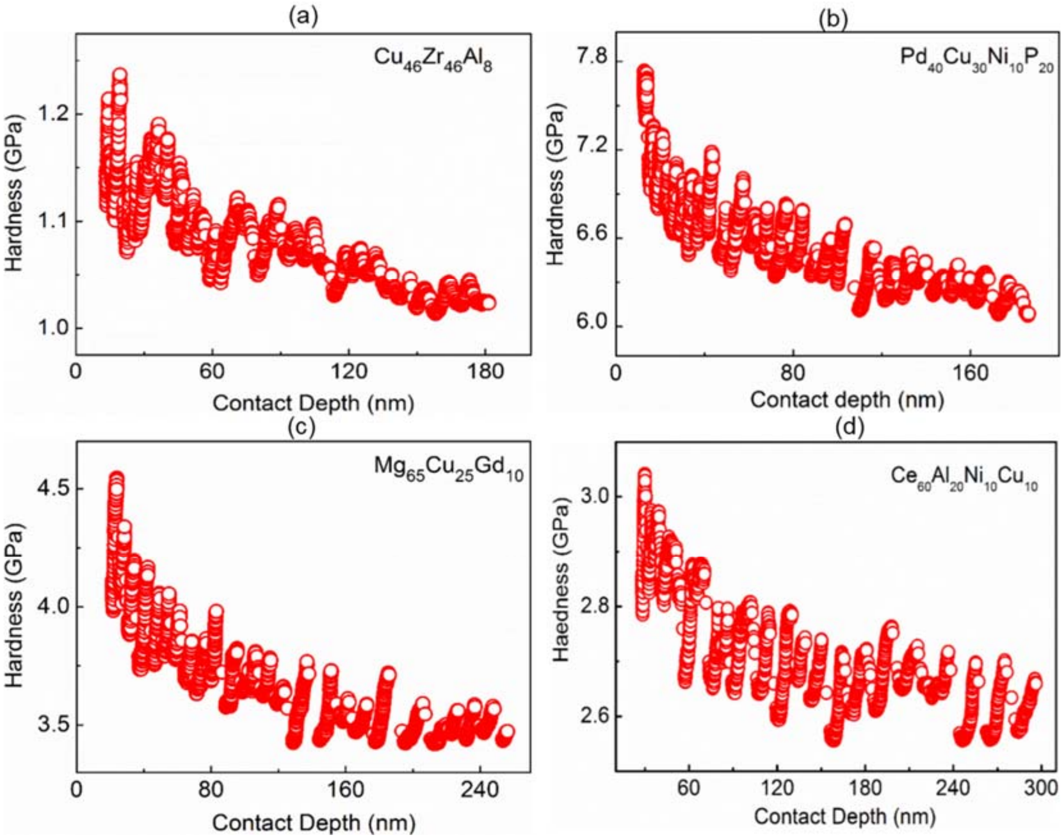

Supplementary Figure 5. The depth dependence of nanohardness for (a) $Cu_{46}Zr_{46}Al_8$ (b) $Pd_{40}Cu_{30}Ni_{10}P_{20}$ (c) $Mg_{65}Cu_{25}Gd_{10}$ (d) $Ce_{60}Al_{20}Ni_{10}Cu_{10}$ MGs obtained at nanoDMA experiments.

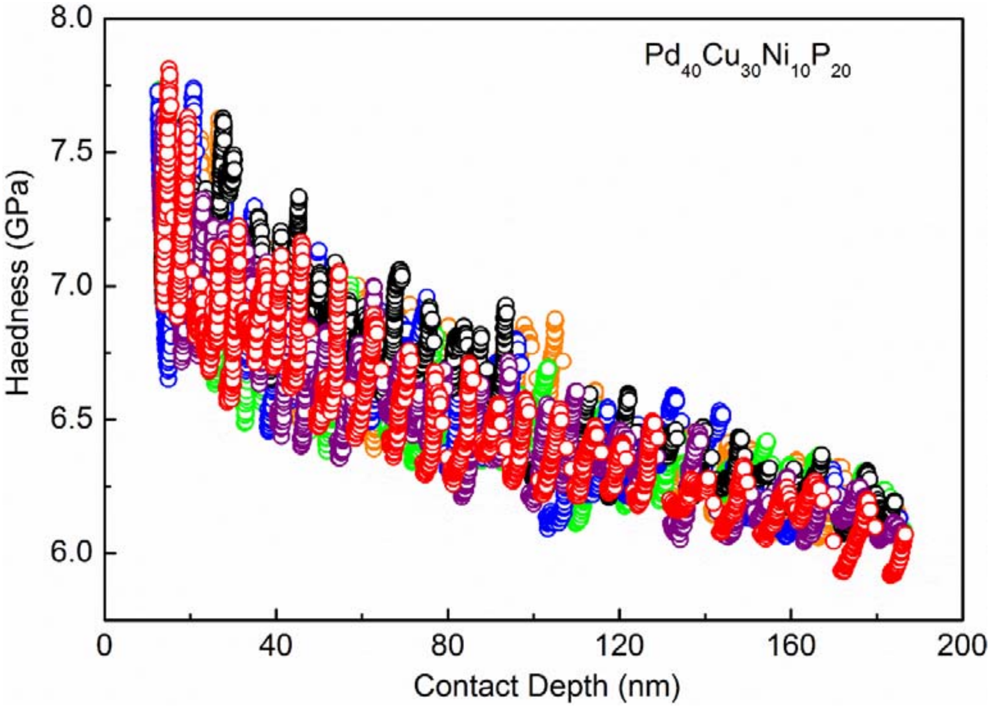

Supplementary Figure 6. Hardness data for $Pd_{40}Cu_{30}Ni_{10}P_{20}$ MG ribbon obtained in six measurements in nanoDMA experiments.

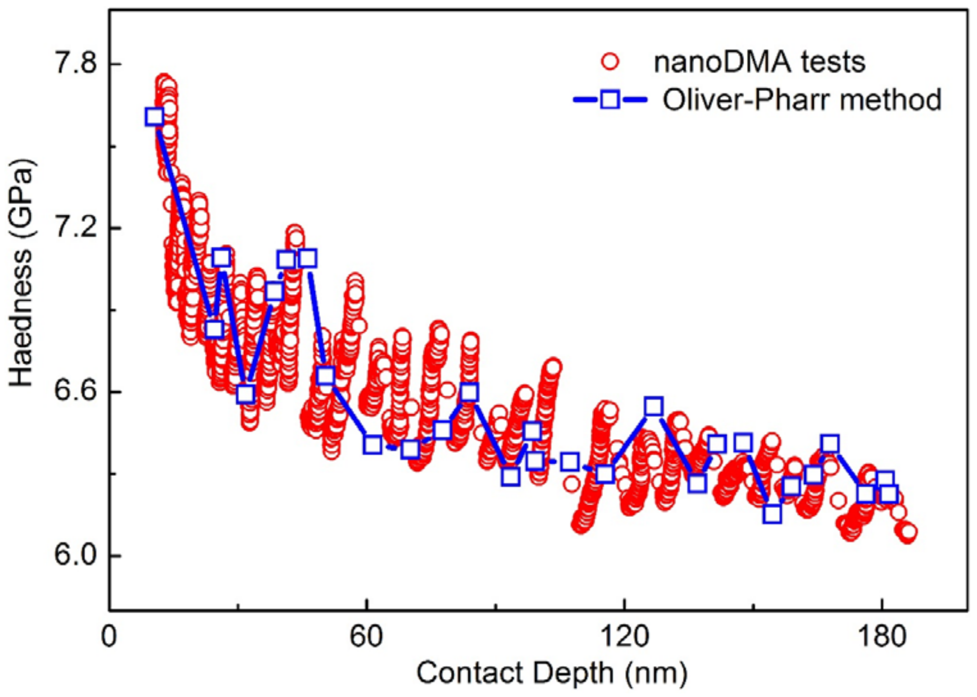

Supplementary Figure 7. Hardness data for the $Pd_{40}Cu_{30}Ni_{10}P_{20}$ MG obtained from the dynamic and normal indentation tests.

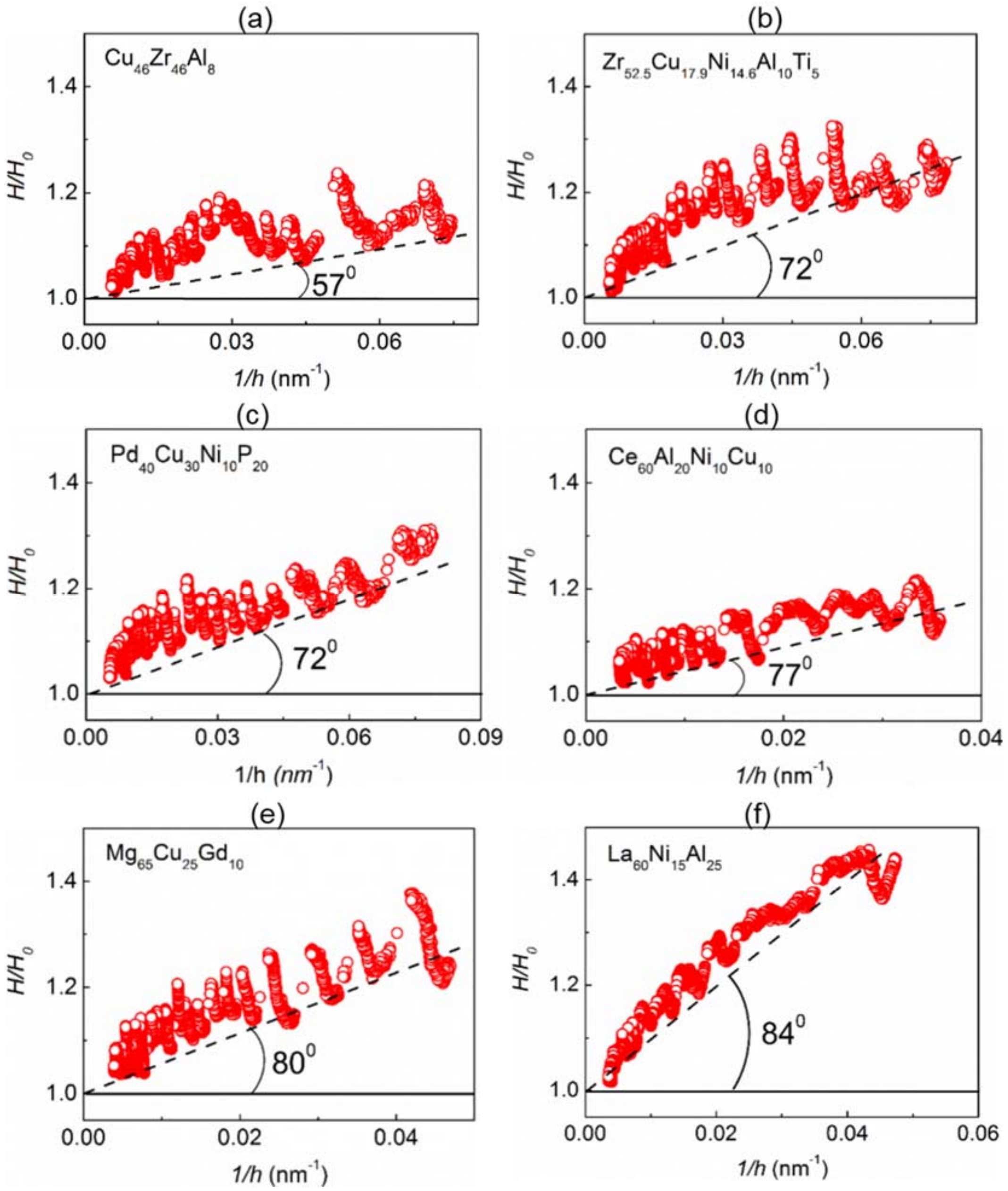

Supplementary Figure 8. Normalized Hardness (*H/H_0*) versus the reciprocal of contact depth (*1/h*) for (a) $Cu_{46}Zr_{46}Al_8$, (b) $Zr_{52.5}Cu_{17.9}Ni_{14.6}Al_{10}Ti_5$, (c) $Pd_{40}Cu_{30}Ni_{10}P_{20}$, (d) $Ce_{60}Al_{20}Ni_{10}Cu_{10}$, (e) $Mg_{65}Cu_{25}Gd_{10}$ and (f) $La_{60}Ni_{15}Al_{25}$ MGs obtained in nanoDMA tests. *H_0* is the bulk hardness. The angles differentiate the indentation size effect in different MGs and larger angles correspond to stronger indentation size effect.

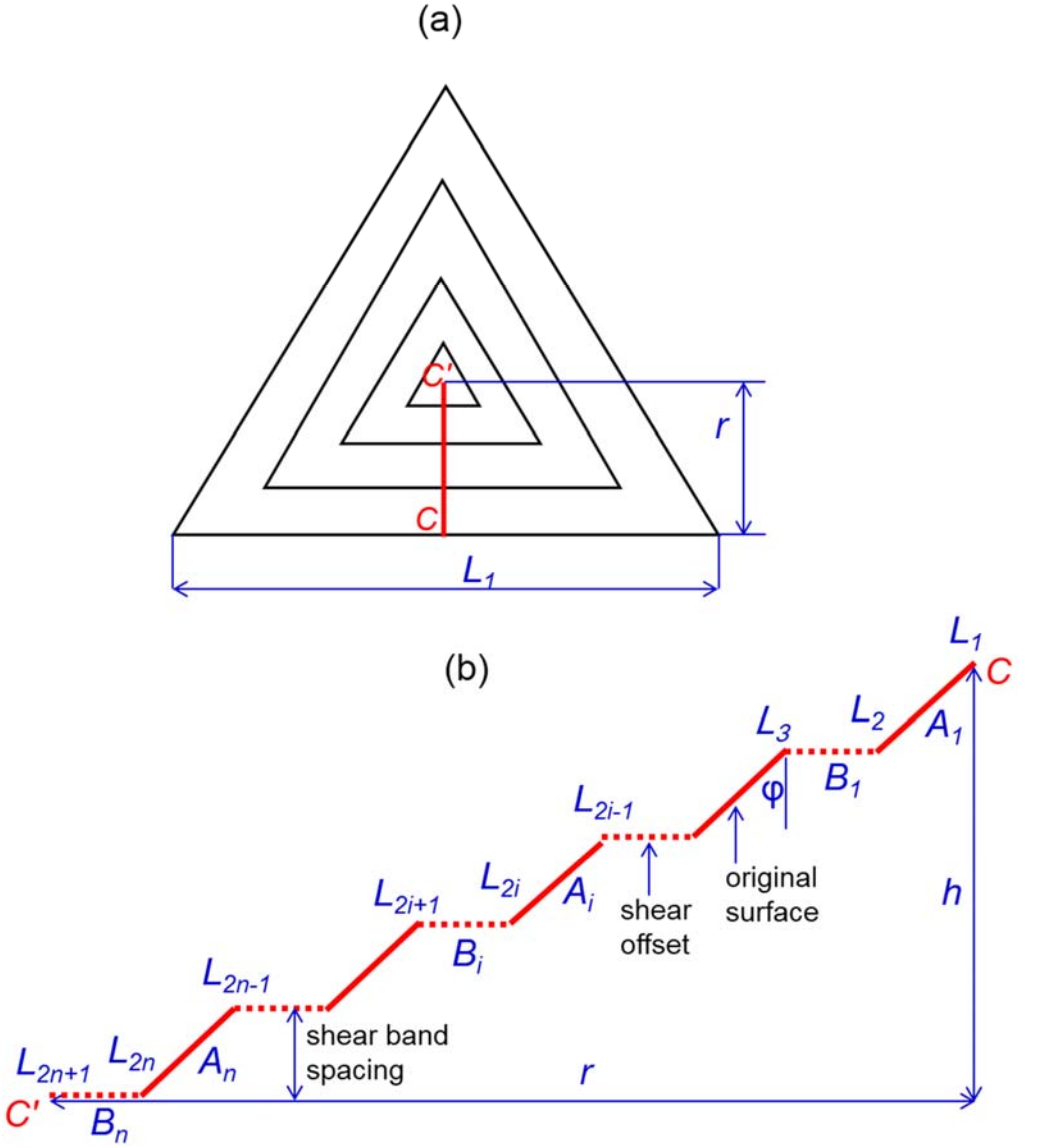

Supplementary Figure 9. (a) The top view of the indentation impression in the indenter face and the deformation is idealized as triangular loops of shear bands. (b) The cross-sectional view along the line *CC′*, in which L_i represents the length of the triangle side for each shear band loop and A_i, B_i represents areas of stretched original surface and shear offset-induced new surface of each shear band loop. *h* is the indentation depth and *r* is the length of *CC′* in Fig.9(a).

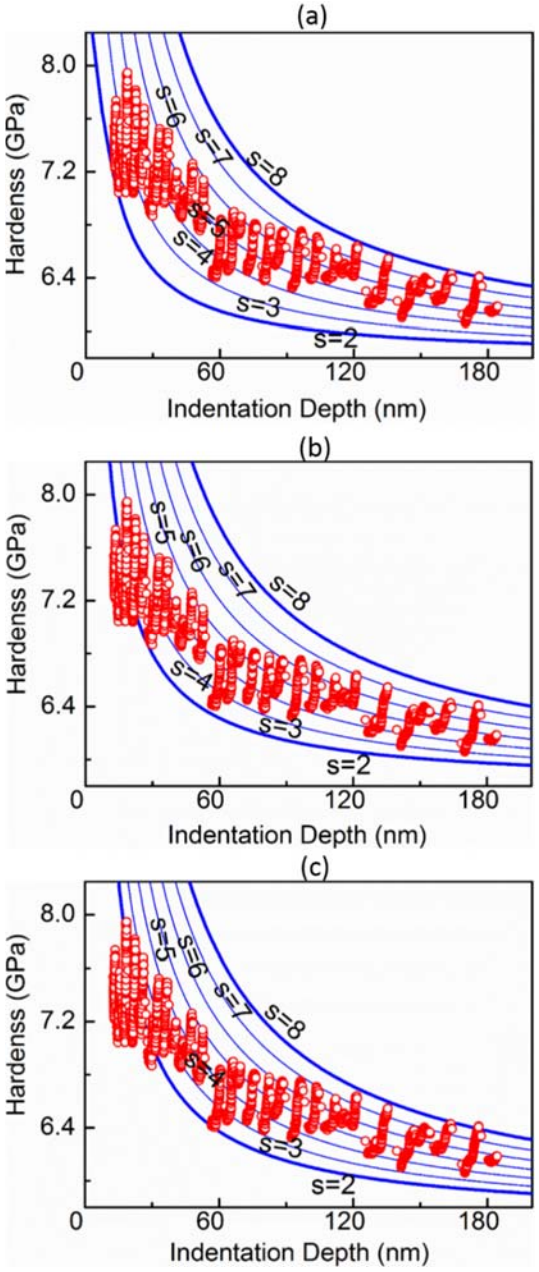

Supplementary Figure 10. The experimental hardness data (red circles) and corresponding theoretical fitting (blue lines) of the DS model for $Zr_{52.5}Cu_{17.9}Ni_{14.6}Al_{10}Ti_5$ MG with (a) γ_s = 1(J/m^2), γ_e = 70(J/m^2), (b) γ_s = 5(J/m^2), γ_e = 70(J/m^2) and (c) γ_s = 10(J/m^2), γ_e = 70(J/m^2) under the same condition that H_0=5.8GPa, t=7.3nm, s=2-8nm.

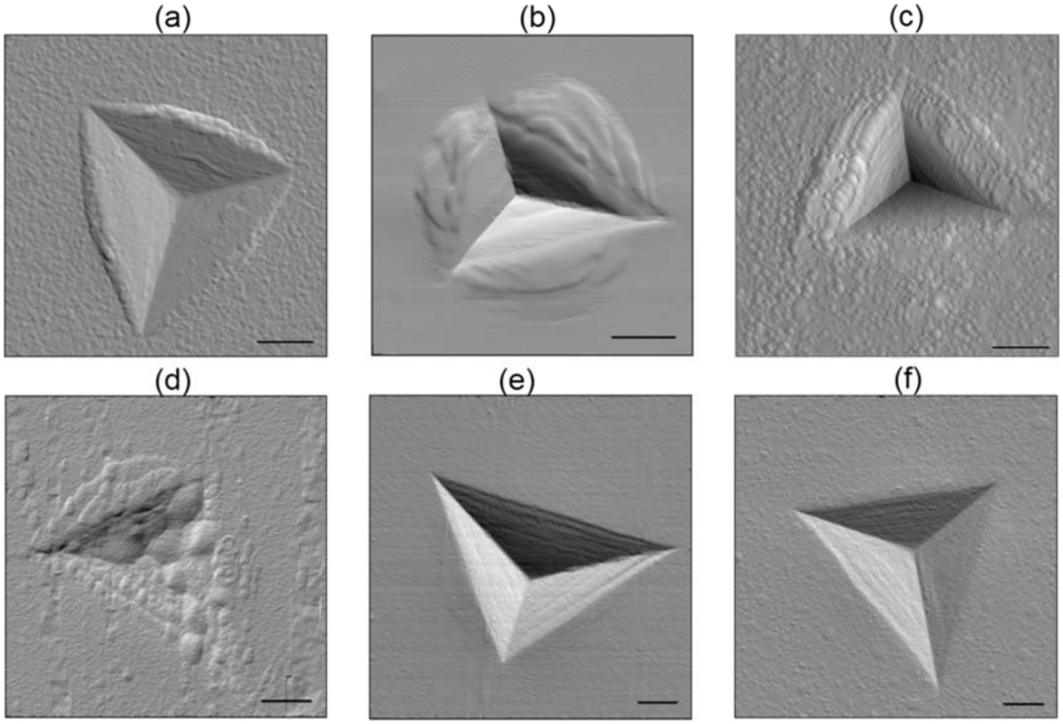

Supplementary Figure 11. Representative atomic force microscopy images of indentation impressions for (a) $Cu_{46}Zr_{46}Al_8$, (b) $Zr_{52.5}Cu_{17.9}Ni_{14.6}Al_{10}Ti_5$, (c) $Pd_{40}Cu_{30}Ni_{10}P_{20}$, (d) $Mg_{65}Cu_{25}Gd_{10}$, (e) $Ce_{60}Al_{20}Ni_{10}Cu_{10}$ and (f) $La_{60}Ni_{15}Al_{25}$ obtained at the indentation load of 8mN. The scale bars are 500nm. All the AFM images are illuminated for clear viewing.

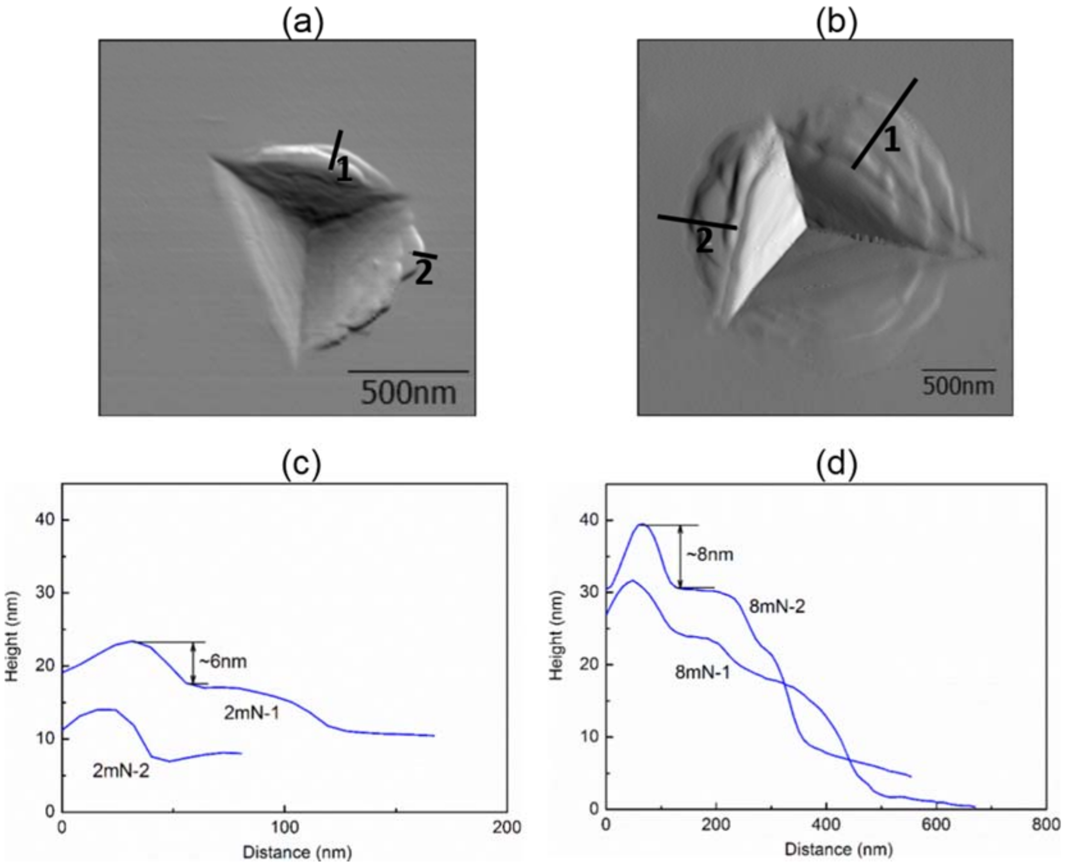

Supplementary Figure 12. Atomic force microscopy images of indentation impressions for $Zr_{52.5}Cu_{17.9}Ni_{14.6}Al_{10}Ti_5$ MG at indentation load of (a) 2mN and (b) 8mN. (c) and (d) are the cross-sectional profiles of black lines indicated in a and b. shear offset values are also indicated in c and d.

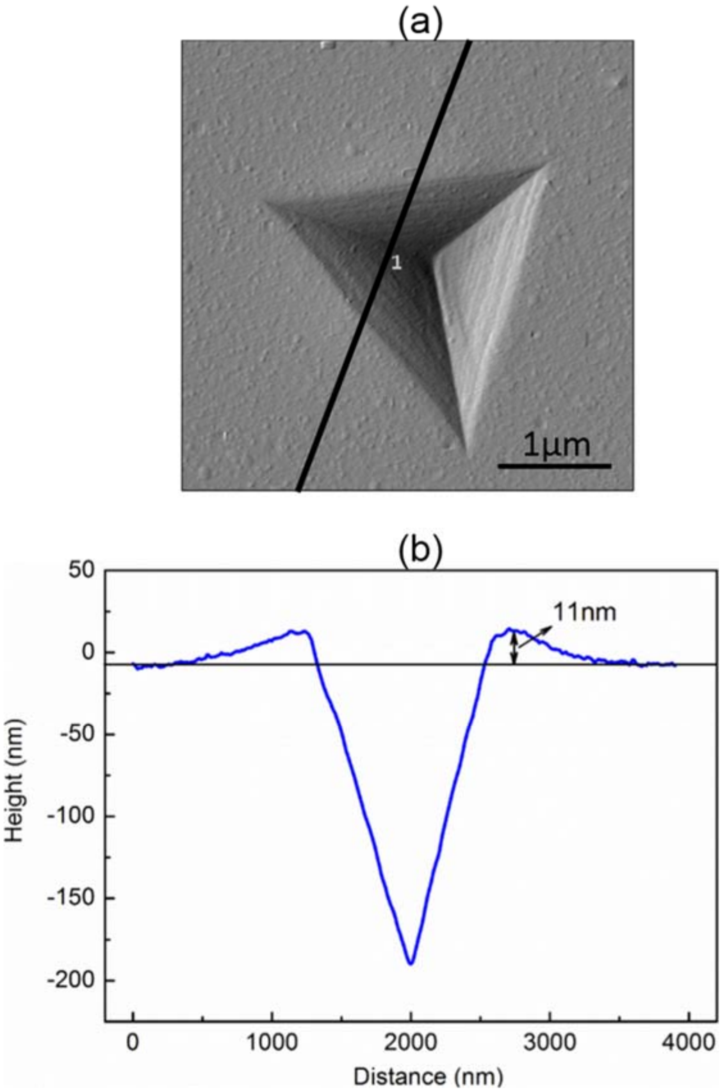

Supplementary Figure 13. (a) The atomic force microscopy images of indentation impressions for $La_{60}Ni_{15}Al_{25}$ MG at indentation load of 8mN (b) The cross-sectional profiles of the black line indicated in Fig.a and the pile up is clearly seen.

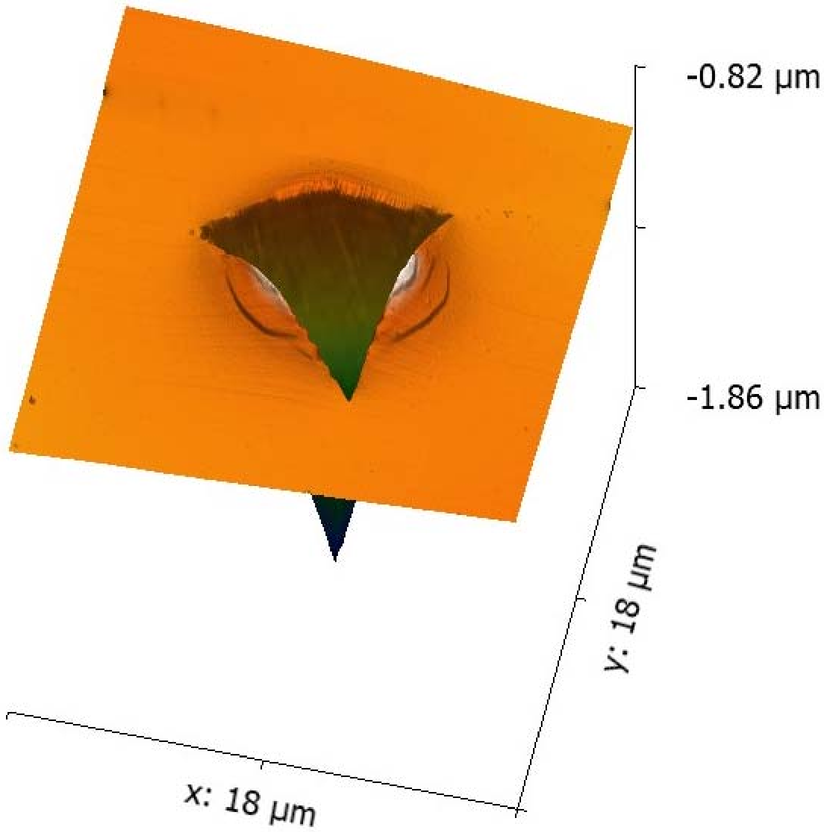

Supplementary Figure 14. The atomic force microscopy image of the indentation impression for $La_{60}Ni_{15}Al_{25}$ MG at indentation load of 80mN.

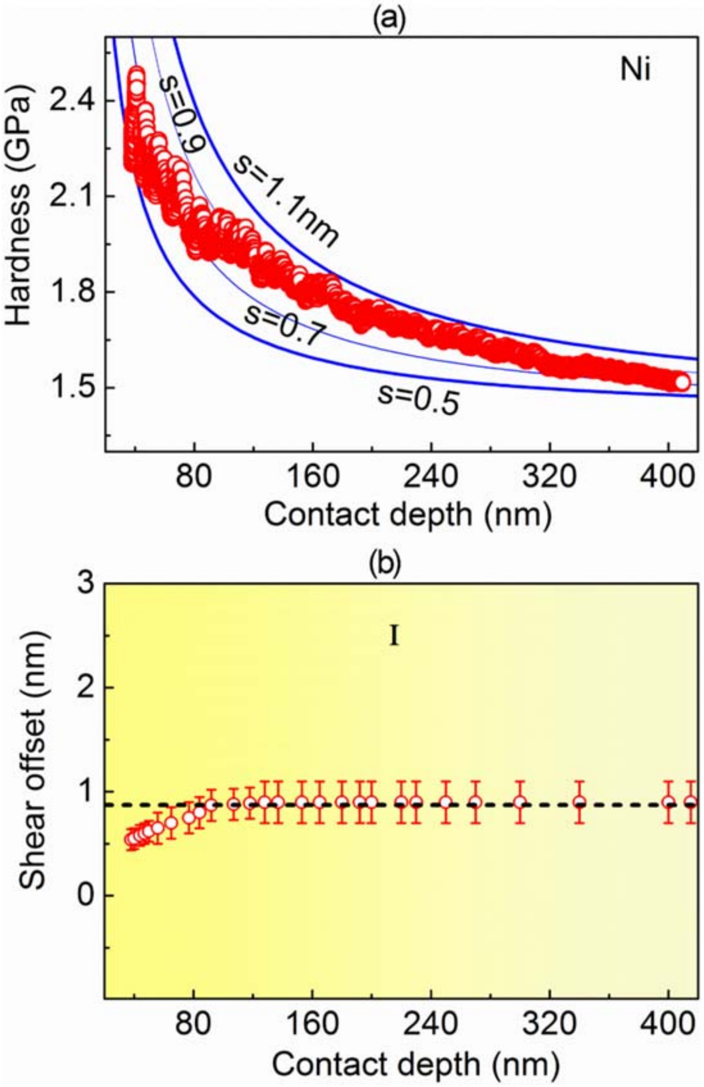

Supplementary Figure 15. (a**)** The experimental nanohardness data (red circles) and corresponding theoretical fitting (blue lines) of the DS model, in which s represents the value of shear offset. (b) The depth dependence of shear offset of the fitting results shown in the top panel. The fitting parameters: γ_e = 50(J/m^2), γ_s = 1(J/m^2), H_0=1.4GPa, t=1nm, s=0.5-1.1nm. The black dotted line is drawn for eye guides.

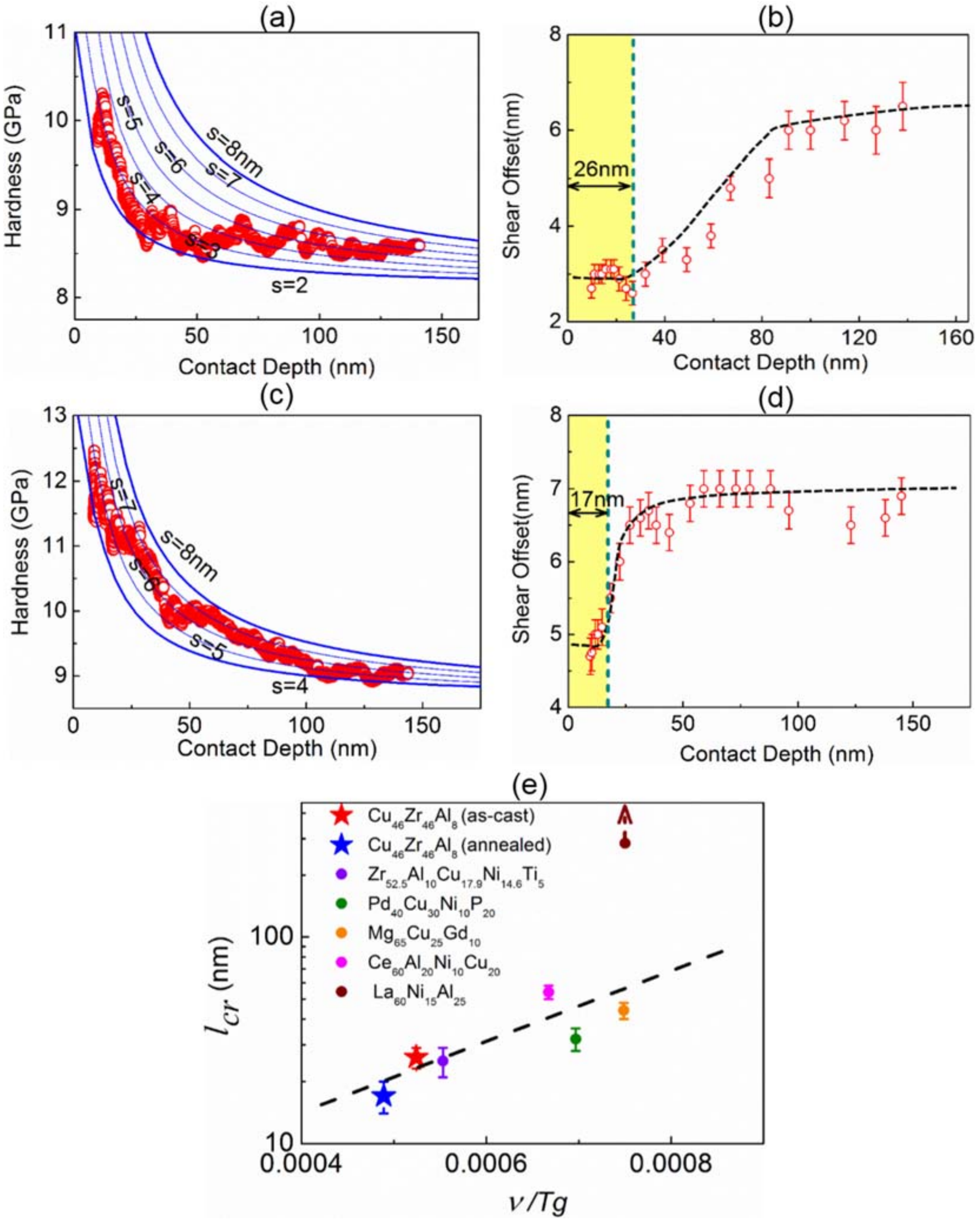

Supplementary Figure 16. The fitting results of the DS model for as-cast (a-b), and annealed (c-d) $Cu_{46}Zr_{46}Al_8$ MGs. The left figures show the experimental hardness data (red circles) and corresponding theoretical fitting (blue lines) of the DS model. The right figures are depth dependence of shear offset of the fitting results in the left figures. Figure e shows the relationship of l_{cr} versus υ/T_g. The black dotted lines are drawn for eye guides.

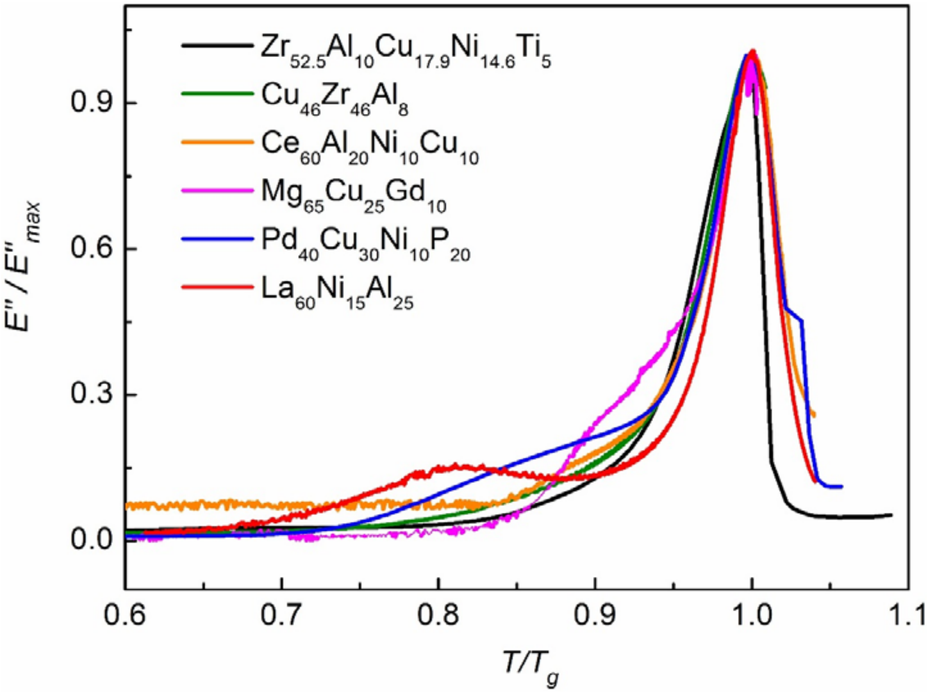

Supplementary Figure 17. Temperature-dependent loss modulus *E/ E″* of the six MGs measured at 4 Hz at a constant heating rate of 3K min^{-1}. The temperature is scaled by glass transition point (T_g) and the E'' is normalized by the corresponding maximum at T_g.

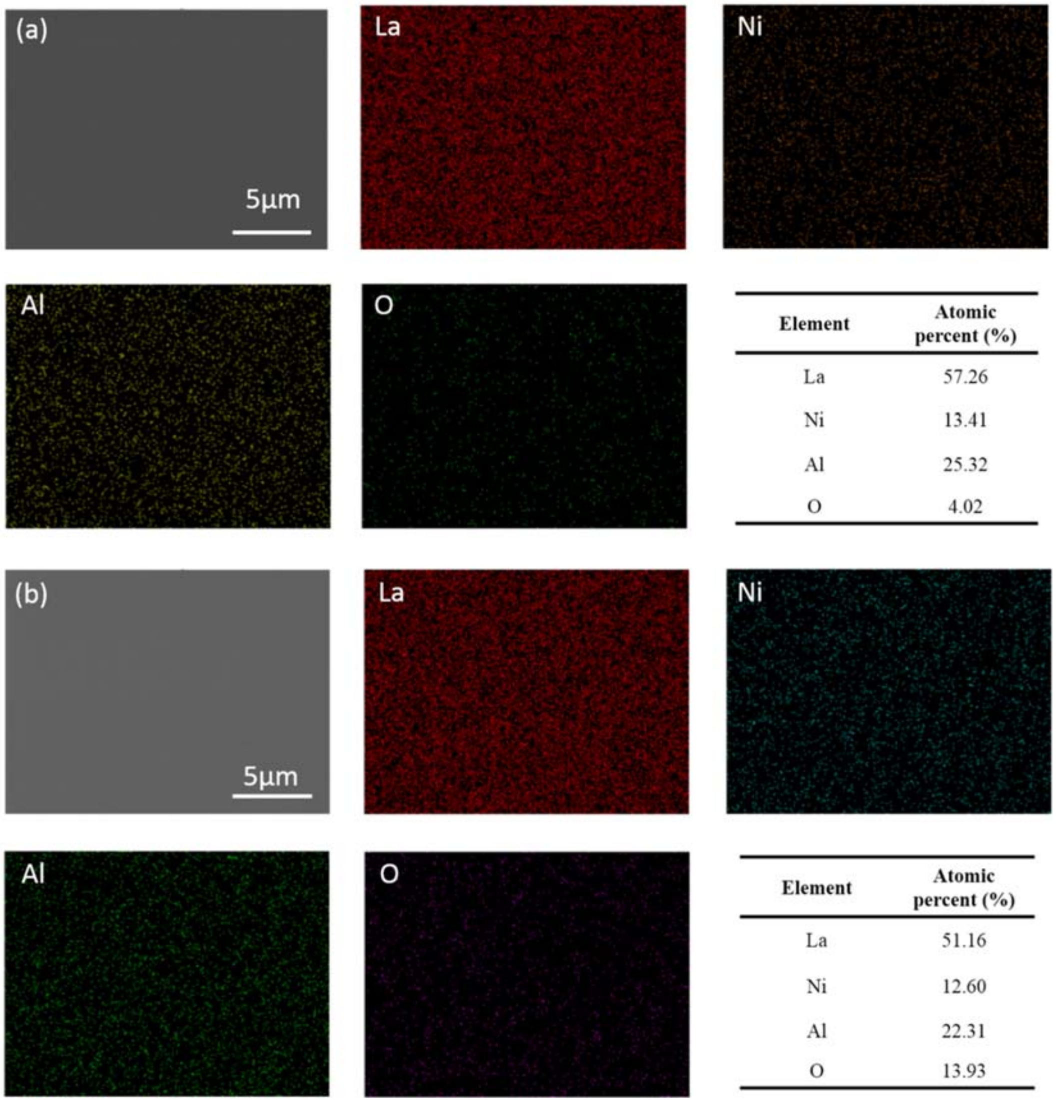

Element	Atomic percent (%)
La	57.26
Ni	13.41
Al	25.32
O	4.02

Element	Atomic percent (%)
La	51.16
Ni	12.60
Al	22.31
O	13.93

Supplementary Figure 18. The energy dispersive X-ray (EDX) characterization of the (a) as-spun and (b) oxidized $La_{60}Ni_{15}Al_{25}$ ribbons (exposed in air for one month) as well as the atomic percent at each state.

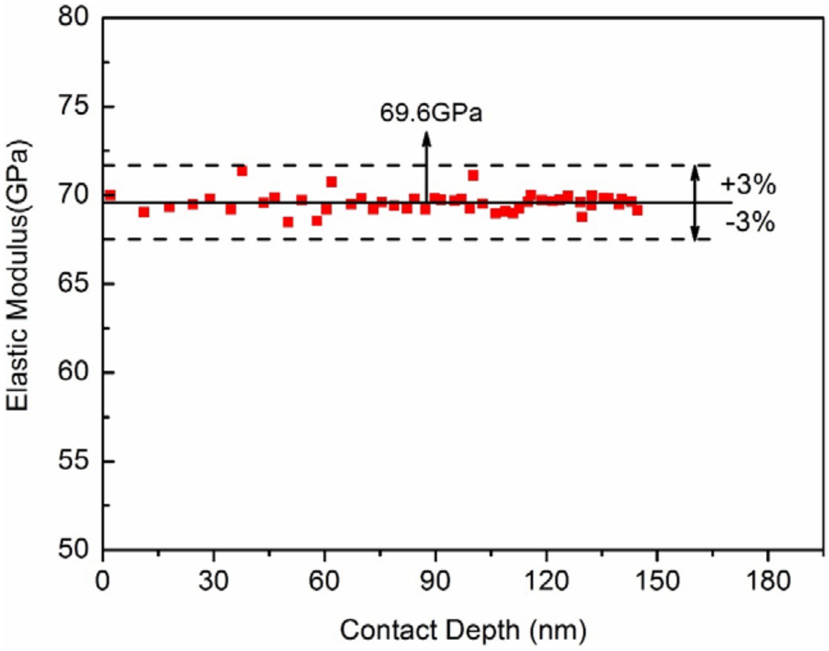

Supplementary Figure 19. Calibrated modulus of quartz obtained using nanoindentation with peak loads from 20μN to 8000μN.

Supplementary Tables

Supplementary Table 1. The fitting parameters of the dissipation energy (γ_e), shear band spacing (t), bulk hardness (H_0) and surface stress (γ_s) in the DS model. For the six MGs, the fitting yields about the same value of 1~10 (J/m^2) for surface stress. For comparison, the table also lists the values of yield stress[1-4] (σ_y) for the six MGs, hardness at the indentation depth of 30nm and the load corresponding to the first obvious pop-in in load-displacement curves.

	γ_e (J/m^2)	t (nm)	H_{max} (GPa)	H_0 (GPa)	$3\sigma_y$ (GPa)	$P_{pop\text{-}in}$ (μN)
$Cu_{46}Zr_{46}Al_8$	70±20	7.5	6.96±0.10	6.11	5.79	161±46
$Zr_{52.5}Cu_{17.9}Ni_{14.6}Al_{10}Ti_5$	80±20	7.3	6.83±0.13	5.84	5.52	174±59
$Pd_{40}Cu_{30}Ni_{10}P_{20}$	80±20	7.1	6.73±0.12	5.71	5.25	182±56
$Mg_{65}Cu_{25}Gd_{10}$	60±10	5.6	4.04±0.16	3.31	2.94	138±42
$Ce_{60}Al_{20}Ni_{10}Cu_{10}$	80±10	6.0	2.93±0.14	2.47	2.4	181±25
$La_{60}Ni_{15}Al_{25}$	90±10	6.1	3.72±0.09	2.71	2.82*	196±24

* σ_y is estimated using the equation $\sigma_y \approx 0.02E$ [1], in which E is elastic modulus.

Supplementary Table 2. Parameters used in the calculation of shear band spacing. T_g is glass transition point, M molar weight, G shear modulus[1,5,6] and ρ material density[1,6].

	T_g (K)	M (kg/mol)	G (GPa)	ρ (g/cm^3)
$Cu_{46}Zr_{46}Al_8$	709	7.335	34.3	7.076
$Zr_{52.5}Cu_{17.9}Ni_{14.6}Al_{10}Ti_5$	669	7.293	32.3	6.730
$Pd_{40}Cu_{30}Ni_{10}P_{20}$	564	7.370	35.5	9.259
$Mg_{65}Cu_{25}Gd_{10}$	414	4.741	19.3	3.794
$Ce_{60}Al_{20}Ni_{10}Cu_{10}$	421	10.169	14.3*	6.669*
$La_{60}Ni_{15}Al_{25}$	471	9.889	17	5.825

* G and ρ are taken as the value of composition $Ce_{60}Al_{15}Ni_{15}Cu_{10}$ from [4] and [1].

Supplementary Table 3. Fragility[1,7-10] (*m*), elastic modulus[1] (*E*) and Poisson's ratio[1,5] (υ) for the six MGs.

	m	*E*	*υ*
$Cu_{46}Zr_{46}Al_8$	43	93.7	0.366
$Zr_{52.5}Cu_{17.9}Ni_{14.6}Al_{10}Ti_5$	49.7	88.6	0.37
$Pd_{40}Cu_{30}Ni_{10}P_{20}$	59	99.8	0.393
$Mg_{65}Cu_{25}Gd_{10}$	41	50.6	0.31
$Ce_{60}Al_{20}Ni_{10}Cu_{10}$	32**	48*	0.281*
$La_{60}Ni_{15}Al_{25}$	43	47	0.353**

* *E* and υ are taken as the value of composition $Ce_{60}Al_{15}Ni_{15}Cu_{10}$ from Ref [1].

** *m* and υ are calculated using the equation $m = \frac{39(1-\upsilon)}{2(1-2\upsilon)}$ according to the Ref [1].

Supplementary Table 4. Glass transition temperature (T_g), shear modulus (G), density (ρ) and Poisson's ratio (υ) used in the annealing experiments. Shear band spacing (t), bulk hardness (H_0) and dissipation energy (γ_e) used in the DS model are also listed in this table.

	T_g (K)	G (GPa)	ρ (g/cm^3)	υ	t (nm)	H_0 (GPa)	γ_e (J/m^2)
$Cu_{46}Zr_{46}Al_8$ (as-cast)	702	34.4	7.076	0.368	7.5	8.1	60
$Cu_{46}Zr_{46}Al_8$ (annealed)	728	36.1	7.098	0.356	9.0	8.6	80

Supplementary Notes

Supplementary Note 1

In Supplementary Fig.9, $h = nt, r = n\times(s + t\times tan\varphi)$ and $L_1 = 2\sqrt{3}r$ in which h is the indentation depth in the loading portion, n is number of shear bands, s is shear offset and t is shear band spacing. Then,

$$\frac{L_{2i}}{L_1} = \frac{(n-i)(s+t\times tan\varphi)+s}{r},\ L_{2i} = \frac{r - is + t\times tan\varphi + s}{r}L_1$$

$$\frac{L_{2i-1}}{L_1} = \frac{n-(i-1)}{n},\ L_{2i-1} = \frac{n-i+1}{n}L_1$$

$$A_i = \frac{\delta}{2cos\varphi}\times L_{2i} + L_{2i-1} \quad B_i = \frac{s}{2}\times L_{2i} + L_{2i+1}$$

$$A = \sum_{i=1}^{n} A_i = \frac{t}{2cos\varphi}\times\sum_{i=1}^{n}\left(\frac{r-(s+t\times tan\varphi)+s}{r}L_1 + \frac{n-i+1}{n}L_1\right)$$

$$= \frac{\sqrt{3}h}{cos\varphi}\times(s + ns + nt\times tan\varphi)$$

$$B = \sum_{i=1}^{n} B_i = \frac{s}{2}\times\sum_{i=1}^{n}\left(\frac{r-(s+t\times tan\varphi)+s}{r}L_1 + \frac{n-i}{n}L_1\right)$$

$$= \sqrt{3}ns\times s + (n-1)\times(s+t\times tan\varphi)$$

$$\frac{\delta A}{\delta h} = \frac{\sqrt{3}}{cos\varphi}\times(s + 2ns + 2htan\varphi)$$

$$\frac{\delta B}{\delta h} = \frac{\sqrt{3}s}{t}\times s + \left(\frac{2h}{t} - 1\right)\times(s + t\times tan\varphi)$$

For a Berkovich indenter, $\varphi = 65.3^0$

$$P\delta h = H_0\delta V + \gamma_s\delta A + \gamma_e\delta B$$

$$H = \frac{P}{\alpha h^2} = H_0 + \gamma_e\times\frac{3\sqrt{3}s}{\alpha h^2}\left(\frac{2h}{t}tan\varphi - tan\varphi + \frac{2hs}{t^2}\right) + \gamma_s\times\frac{3\sqrt{3}}{\alpha h^2 cos\varphi}\left(2htan\varphi + \frac{2hs}{t} + s\right)$$

Supplementary Note 2

To calculate the shear band spacing (t), the equation[11] of $t = \sqrt[3]{\left[12\varphi_c\left(\tau y - \beta\sigma N\right)/\rho\dot{\gamma}^2)\right]}$ is used, in which τ_y is flow stress, σ_N normal stress, $\dot{\gamma}$ shear strain rate, ρ the density of the material, β a constant value and φ_c the critical shear displacement at which shear band is fully mature. In nanoindentation tests, we consider φ_c is approximately 10nm and $\beta\sigma_N$ is negligible since it is very small compared with τ_y[12,13]. In Chen's work[14], $\tau_y / \rho = \frac{3R \times \left(T_g - T\right)}{M\gamma_0}$, in which R is gas constant, T_g glass transition temperature, T room temperature, M molar weight and $\gamma_0 = 1$. Shear strain rate is calculated through the equation $\dot{\gamma} = \frac{\mu}{\lambda}$, in which μ is shear band velocity and λ is shear band thickness (about 10nm[15]). According to previous studies[16], $\mu = 0.9c_t = 0.9\sqrt{\frac{G}{\rho}}$, in which c_t is the velocity of the transverse sound wave, G is shear modulus and ρ is material density. Parameters of T_g, M, G and ρ are listed in Supplementary Table 1.

Supplementary Note 3

To study the annealing effect on the size-controlled transition behavior of shear banding, we performed nanoDMA experiments with annealed MGs and calculate the critical length l_{cr} based on our DS model. In the annealing experiment, glassy alloy rod with the composition of $Cu_{46}Zr_{46}Al_8$ with 5mm in diameter was prepared by copper mold suck-casting method and the annealed samples were obtained by annealing the cast samples encapsulated in a quartz crucible with vacuum of 10^{-4} Pa. at $0.88T_g$ (618K) for 32 hours. The amorphous nature of MGs before and after annealing was confirmed by X-ray diffraction[17]. Glass transition temperatures were obtained in DSC measurements and Poisson's ratio and modulus were calculated by using acoustic velocities obtained from the acoustic measurements[1]. The density ρ was measured by Archimedes' principle. These parameters are listed in Supplementary Table 4. Hardness obtained in nanoDMA experiments and the fitting results of DS model are shown in Supplementary Fig.16. It can be seen that l_{cr} decreases after annealing.

Supplementary References

1 Wang, W. H. The elastic properties, elastic models and elastic perspectives of metallic glasses. *Prog. Mater. Sci.* **57**, 487-656, (2012).

2 Li, W. H., Shin, K., Lee, C. G., Wei, B. C. & Zhang, T. H. Simple phenomenological determination of contact stiffness and elastic modulus of Ce-based bulk metallic glasses through nanoindentation. *Appl. Phys. Lett.* **90**, 171928, (2007).

3 Liu, Z. Q., Qu, R. T. & Zhang, Z. F. Elasticity dominates strength and failure in metallic glasses. *J. Appl. Phys.* **117**, 014901, (2015).

4 Madge, S. V. Toughness of Bulk Metallic Glasses. *Metals* **5**, 1279-1305, (2015).

5 Liu, S. T., Wang, Z., Peng, H. L., Yu, H. B. & Wang, W. H. The activation energy and volume of flow units of metallic glasses. *Scr. Mater.* **67**, 9-12, (2012).

6 Fornell, J., Surinach, S., Baro, M. D. & Sort, J. Unconventional elastic properties, deformation behavior and fracture characteristics of newly developed rare earth bulk metallic glasses. *Intermetallics* **17**, 1090-1097, (2009).

7 Wei, S. *et al.* Linking structure to fragility in bulk metallic glass-forming liquids.

Appl. Phys. Lett. **106**, 181901, (2015).
8 Wang, Z., Yu, H. B., Wen, P., Bai, H. Y. & Wang, W. H. Pronounced slow beta-relaxation in La-based bulk metallic glasses. *J. Phys-Condens. Mat.* **23**, 142202, (2011).
9 Novikov, V. N. & Sokolov, A. P. Correlation of fragility and Poisson's ratio: Difference between metallic and nonmetallic glass formers. *Phys. Rev. B* **74**, 064203, (2006).
10 Perera, D. N. Compilation of the fragility parameters for several glass-forming metallic alloys. *J. Phys-Condens. Mat.* **11**, 3807-3812, (1999).
11 Zhang, H. W., Maiti, S. & Subhash, G. Evolution of shear bands in bulk metallic glasses under dynamic loading. *J. Mech. Phys. Solids* **56**, 2171-2187, (2008).
12 Lowhaphandu, P., Montgomery, S. L. & Lewandowski, J. J. Effects of superimposed hydrostatic pressure on flow and fracture of a Zr-Ti-Ni-Cu-Be bulk amorphous alloy. *Scr. Mater.* **41**, 19-24, (1999).
13 Lewandowski, J. J. & Lowhaphandu, P. Effects of hydrostatic pressure on the flow and fracture of a bulk amorphous metal. *Philos. Mag. A* **82**, 3427-3441, (2002).
14 Liu, Y. H. *et al.* Thermodynamic Origins of Shear Band Formation and the Universal Scaling Law of Metallic Glass Strength. *Phys. Rev. Lett.* **103**, 065504, (2009).
15 Zhang, Y. & Greer, A. L. Thickness of shear bands in metallic glasses. *Appl. Phys. Lett.* **89**, 071907, (2006).
16 Miracle, D. B., Concustell, A., Zhang, Y., Yavari, A. R. & Greer, A. L. Shear bands in metallic glasses: Size effects on thermal profiles. *Acta Mater.* **59**, 2831-2840, (2011).
17 Xue, R. J., Zhao, L. Z., Pan, M. X., Zhang, B. & Wang, W. H. Correlation between density of metallic glasses and dynamic fragility of metallic glass-forming liquids. *J. Non-Cryst.Solids* **425**, 153-157, (2015).
